# Analyses of unpredictable properties of a wind-driven triboelectric random number generator

**DOI:** 10.1038/s41598-023-43894-1

**Published:** 2023-10-03

**Authors:** Moon-Seok Kim, Il-Woong Tcho, Yang-Kyu Choi

**Affiliations:** 1 School of Electrical Engineering, Korea Advanced Institute of Science and Technology (KAIST), 291 Daehak-ro, Yuseong-gu, Daejeon, 34141 Republic of Korea; 2 Department of Semiconductor System Engineering, Hanbat National University, 125 Dongseo-daero, Yuseong-gu, Daejeon, 31538 Republic of Korea

**Keywords:** Energy harvesting, Electrical and electronic engineering, Information technology

## Abstract

Wind-driven triboelectric nanogenerators (W-TENGs) are a promising candidate for an energy harvester because wind itself possesses unexhausted, ubiquitous, and clean properties. W-TENG has also been used as a random number generator (RNG) due to the inherent chaotic properties of wind that is also an entropy source. Thus, a W-TENG which simultaneously generates both power and true random numbers with a two-in-one structure, is a wind-driven RNG (W-RNG) like the Janus. However, a root cause of W-RNG unpredictability has not been elucidated. In this work, the unpredictability, which is essential and critical for an RNG, is statistically and mathematically analyzed by auto-correlation, cross-correlation, joint entropy, and mutual information. Even though the overall shape of the total output analog signals from the W-RNG looks like a sinusoidal wave that is not obviously unpredictable, discretized digital signals from the continuous analog output become unpredictable. Furthermore, partial adoption of 4-bit data from 8-bit raw data, with the aid of analog-to-digital converter hardware, further boosts the unpredictability. The W-RNG, which functions as a W-TENG, can contribute to self-powering and self-securing outdoor electrical systems, such as drones, by harvesting energy and generating true random numbers.

## Introduction

Recently, the Internet of Things (IoT) technology has emerged as an innovative paradigm which attains hyper-connectivity of objects, devices, and people^1^. It is estimated that the number of connected devices will grow to 20 billion in the near future^2,3^. In IoT and smart system technology, each device must possess the ability to supply stable energy and to communicate with other devices^4^. Security functions such as confidentiality, integrity, availability, authentication, and non-repudiation are crucial for securing the communications among all connected devices^5–10^. A true random number generator (TRNG), which is one of the promising security primitives based on hardware, plays a significant role in supporting the aforementioned security functions. Thus, the development of a TRNG with a variety of entropy sources is vital to achieving secure IoT technology^11–15^. There were a few reports on devising a TRNG using various entropy sources in nature^16–18^. However, their extraction of true random numbers through post-processing can be a demerit in reducing power consumption. If possible, no use of post-processing is preferred.

Alternatively, a TRNG without post-processing was demonstrated with the aid of a prototyped wind-driven triboelectric nanogenerator (W-TENG)^19^. Their W-TENG based TRNG not only provides energy harvesting but also security functions for communicating systems *e.g.* IoT, a smart grid for an electricity network, and in-flight applications. It produces true random numbers by transferring chaotic wind flow to random electrical signals via triboelectrification. The random electrical signals produced from hardware with a wind entropy source are generated without any post-processing algorithms. The random signals from the W-TENG satisfy the requirements of the NIST SP 800-22B, which is the most widely used standard methodology to evaluate randomness. Previous works have reported that randomness is attributed to the chaotic behaviors of wind^20^. However, intensive and in-depth analyses regarding both the unpredictability of the output signals from the W-TENG and suitability as an entropy source remain insufficient from a theoretical and statistical point of view. It is now crucial to quantify the unpredictable properties to enhance the practicality of W-TENG based TRNG.

In this work, we conducted in-depth analyses of the unpredictability and randomness of the generated random numbers from the W-TENG based TRNG. We examined auto-correlation, cross-correlation, joint entropy, and mutual information, ensuring distinctive security features such as confidentiality, integrity, availability, authentication, and non-repudiation. As a significant advancement compared to our previous work^19^, we installed the W-TENG on a commercial drone, directly harvesting energy from the wind generated by the drone’s rotating blades. We then used this harvested energy to power a light-emitting diode, effectively demonstrating the W-TENG’s capability as an energy harvester. Moreover, our approach for extracting true random numbers diverged from traditional software-based methods reliant on virtual analog-to-digital converters (ADCs). Instead, we sourced these true random numbers directly from the hardware of an ADC connected to the W-TENG, thereby establishing a reliable and hardware-based random number generator. Consequently, the W-TENG based TRNG can contribute to securing and self-powering outdoor electrical systems by harvesting energy and generating true random numbers. Moreover, it can also enhance the security of smart power grids.

## Materials and methods

### Fabrication of wind-driven TENG

For this study, we fabricated another W-TENG with an enclosed thin flip-flop polytetrafluoroethylene (PTFE) film^19^. The thickness of the PTFE film is 0.2 mm. The PTFE film has a small gap between a top and a bottom electrode to guide air flow. This air gap (*h*) acts as an inducer to generate a chaotic vortex from inlet wind. The size of the Al electrodes is 40 mm in length × 40 mm in width, and its thickness is 1 mm^20^. To separate the top and bottom electrode, 4 spacers are employed at each corner of the W-TENG. Each spacer is composed of neodymium magnets, which are vertically stacked^21,22^. The size of the neodymium magnets is 5 mm in length × 5 mm in width, and its thickness is 0.5 mm. Because the size of the magnet spacer is small enough compared to the size of the electrodes, it cannot influence the wind vortex in the air-gap. In each corner, the PTFE film is fixed in between upper two magnets and lower two magnets. The air-gap distance between the top and bottom electrode becomes 2.2 mm because the sum of 0.5 mm × 4 magnets and 0.2 mm PTFE film is 2.2 mm.

### Applied wind pressure

Artificial wind was generated by an air gun with a regulator (SUS316L EP regulator), which is used to control the wind speed for a referenced test. The regulator controls wind pressure, which is ranged from 10 psi (4.3 m/s) to 30 psi (12.8 m/s). In all control experiments, unless specified otherwise in Fig. 7, the artificial wind was maintained at an inlet pressure of 30 psi (12.8 m/s). For actual experiments, a semi-natural wind generated by the rotating wings of a drone was utilized.

## Results and discussion

### Implementation of wind-driven triboelectric random number generator

**Figure 1:**
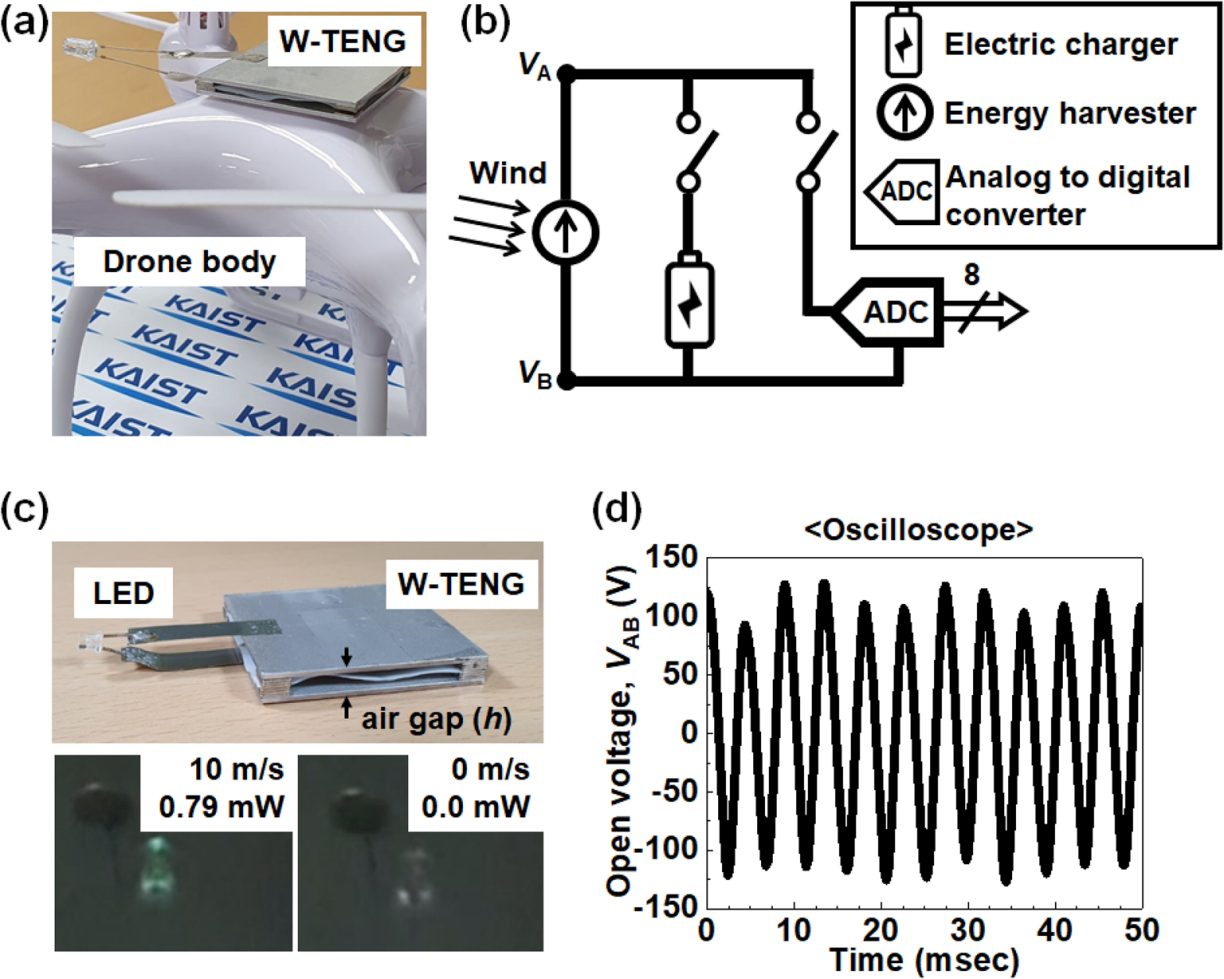
(**a**) Manufactured two-in-one type W-RNG mounted on a commercial drone. (**b**) Electrical configuration of the W-RNG, electric charger, and ADC. (**c**) Optical photographs of turned-on LED with a wind pressure of 30 psi (left) and turned-off LED with 0 psi (right). (**d**) Measured output voltage from the W-RNG with a wind pressure of 30 psi.

Our manufactured W-TENG can simultaneously act as a power generator and a TRNG, with a two-in-one configuration like the Janus. Figure 1a shows the two-in-one type W-TENG mounted on a commercial drone. A light emitting diode (LED) is utilized as an optical indicator to assure that power is generated from the installed W-TENG during flight. Figure S1 exhibits the lighting of the LED, which signifies power generation from the W-TENG on the drone. For the actual experiments, the applied wind pressure to the W-TENG was driven by the rotating wings of the drone. Figure S1a shows the W-TENG with the LED mounted on the drone. It is known that wind pressure is maximal at the center of the drone, which has 4 rotary wings^23,24^; thus, the W-TENG with the LED was installed at the center of the drone. Figure S1b illustrates a brief electric circuit comprised of the W-TENG and the LED. Figure S1c shows an image of the lightened LED, and Fig. S1d displays the close-up view of the inset, denoted by the white dashed line. This figure compares LED images at the turned-on state (left column) and at the turned-off state (right column). As shown in Fig. 1b, in accordance with a purpose, an end user can employ an operational mode: a power generating mode for energy harvesting and an RNG mode for cryptographic communication. When the energy harvesting mode is enabled, the generated output signal is transformed to an electric charger in a drone as a power source for a TENG. When the RNG mode is activated, the analog output voltage signals are converted to 8-bit digital signals that are discretized by a sampling process via an ADC, as an entropy source for an RNG. Figure 1c shows that the LED was connected with two electrodes of the manufactured W-TENG and turned on solely by its generated electricity. For this in-door experiment, controlled wind was set to 30 psi, which is approximately 12.8 m/s, which produced 0.79 mW. The peak power of 0.79 mW was obtained by multiplying output voltage of 149 V and output current of 5.3 μA at resistance of 10 MΩ. Figure 1d shows the measured output voltage between *V*_A_ and *V*_B_ of Fig. 1b with respect to the time evolution. It resembles a sinusoidal wave; however, it is not actually a genuine sine wave. Such sine wave-like behavior is ascribed to the alternating flip-flop actuation of the thin PTFE film between the two electrodes. Pseudo-sine wave behavior, but with unpredictable randomness, is attributed to the chaotic wind vortex in the airgap. In the initial state, the thin PTFE film was located in the middle, which is in between the top and the bottom electrode. When wind is introduced to the small gap of the W-TENG, it flutters upwardly and downwardly between the two electrodes. Physical contact between the PTFE film and the aluminum electrode induces triboelectric effects, which generate electrical energy. Herein the electrical output signal is a continuous analog voltage arising from the iterative flip-flop of the PTFE film. Through the abovementioned ADC hardware, the analog voltage can be converted to a digital signal^25,26^.

**Figure 2:**
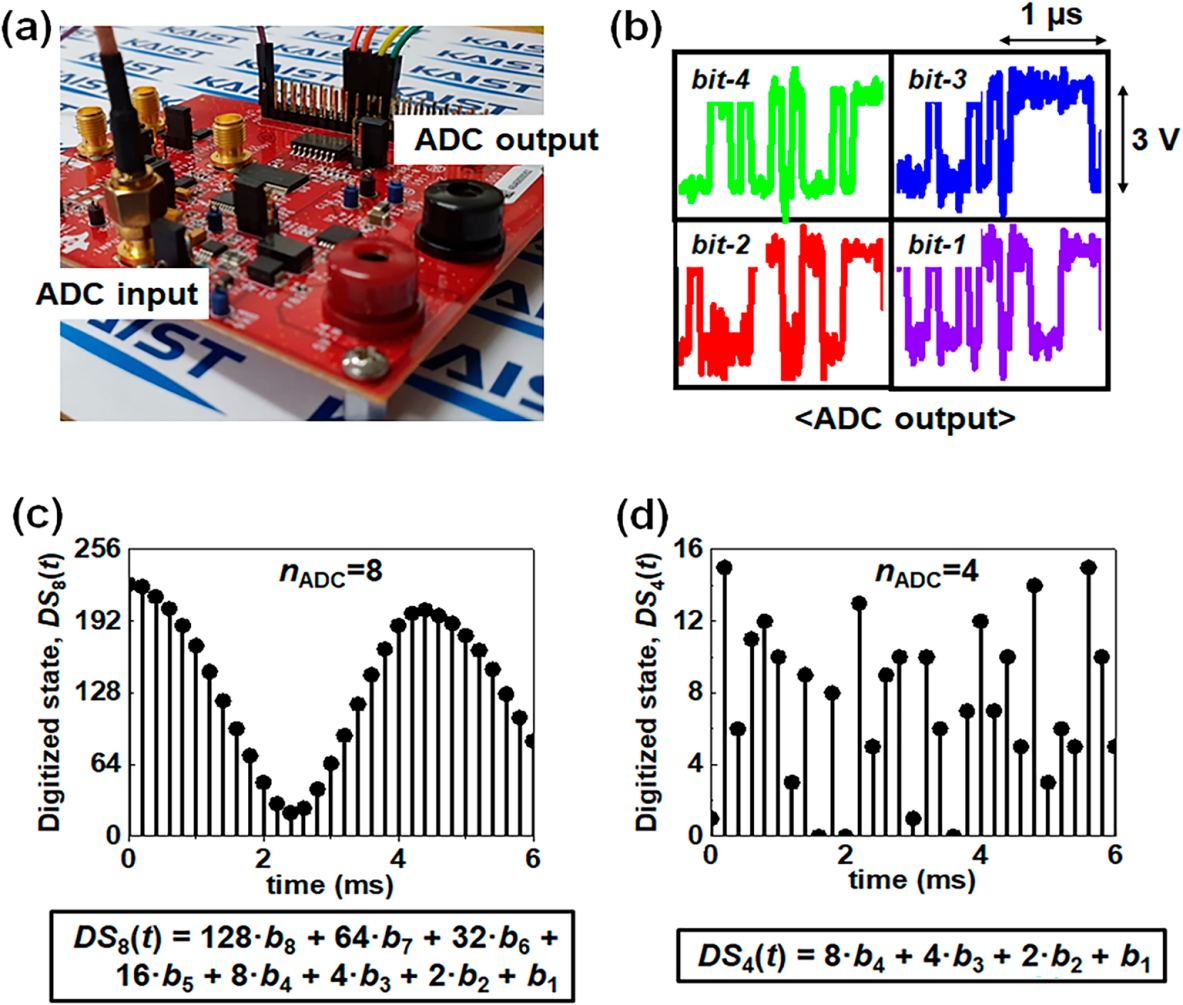
(**a**) Optical photograph of employed ADC test board with the ADC-08100 module. (**b**) Measured output voltage from the ADC test board for *bit-4*, *bit-3*, *bit-2*, and *bit-1*. (**c**) Measured discrete states of *DS*_8_(*t*) ranging from 0 to 255 for signals of *bit-8* to *bit-1*. (**d**) Measured digitized states of *DS*_4_(*t*) ranging from 0 to 15 for signals of *bit-4* to *bit-1*.

Figure 2a exhibits a photograph of an ADC-08100 evaluation module (EVM) for the conversion of the analog-to-digital^27^. In our previous works, such conversion was realized by use of Matlab in a software manner^19,20^. In contrast, the hardware-based data conversion in this work was conducted using the ADC-08100 EVM module mounted on a printed circuit board (PCB) with power consumption at a mW level, as a proof-of-concept for actual random number generation from a W-TENG based TRNG. Such a high level of power consumption is attributed to the extra power consumption of supportive components mounted on the same PCB such as a low-dropout voltage regulator, clock buffer, output buffer, and onboard crystal oscillator. If an ADC is dedicated to a W-TENG based TRNG and constructed as a type of system-on-chip (SoC), its power consumption can be reduced to less than 200 nW, as shown in Table S1^28–30^. In this work, each output pin corresponding to each digital output among the 8-bits is assigned as *bit-8*, *bit-7*, *bit-6*, and so on*,* in the ADC-08100 EVM. The *bit-8* signal refers to the most significant bit (MSB) signal, while the *bit-1* signal indicates the least significant bit (LSB) signal. Figure 2a shows the four conductive cables connected to the ADC output pins for *bit-4*, *bit-3*, *bit-2*, and *bit-1* signals among the 8-bit digital bits. Furthermore, each conductive cable is characterized by an oscilloscope. Figure 2b shows each waveform of the measured voltage from the *bit-4*, *bit-3*, *bit-2*, and *bit-1* signals through the ADC-08100 EVM. The measured *bit-4*, *bit-3*, *bit-2*, and *bit-1* signals are represented as green, blue, red, and purple, respectively. As shown in Fig. 2b, the voltage difference between the high-level and low-level output voltage is 3.0 V in the ADC-08100 EVM.

Herein, ${DS}_{{n}_{\mathrm{ADC}}}(t)$ is defined to show a transient digital state, where *n*_ADC_ is the number of the extracted bit among the 8 output bits in the ADC. It is represented as follows:

$$DS_{{n_{{{\text{ADC}}}} }} \left( t \right) = \mathop \sum \limits_{j = 1}^{{n_{{{\text{ADC}}}} }} 2^{j - 1} \cdot b_{j} { }\left( {n_{{{\text{ADC}}}} = 1, \ldots ,8} \right)$$

where *b*_*j*_ is a bit value for a bit-*j*^th^ signal and *t* is the time evolution. *DS*_8_(*t*) ranges from 0 to 255, while *DS*_4_(*t*) ranges from 0 to 15. The value of *b*_*j*_ is ‘0’ or ‘1’. For example, when the measured bits for each of the 8 bits are 1, 0, 1, 0, 1, 0, 1, and 1 in descending order at *t* = 100 μs, *DS*_8_(100 μs) becomes 179 (2^7^ + 2^5^ + 2^3^ + 2^1^ + 2^0^) and *DS*_4_(100 μs) is 11 (2^3^ + 2^1^ + 2^0^). Figure 2c represents the measured transient digital state of *DS*_8_(*t*) enclosing the 8 output bits and Fig. 2d denotes that of the *DS*_4_(*t*) including 4 output bits. The digitized state of the *DS*_8_(*t*) seems to follow a periodic and regular pattern like a sinusoidal wave. In contrast, the digitized state of the *DS*_4_(*t*) conforms to a sporadic and irregular pattern unlike a sinusoidal wave.

### In-depth analysis for unpredictability of random numbers

**Figure 3:**
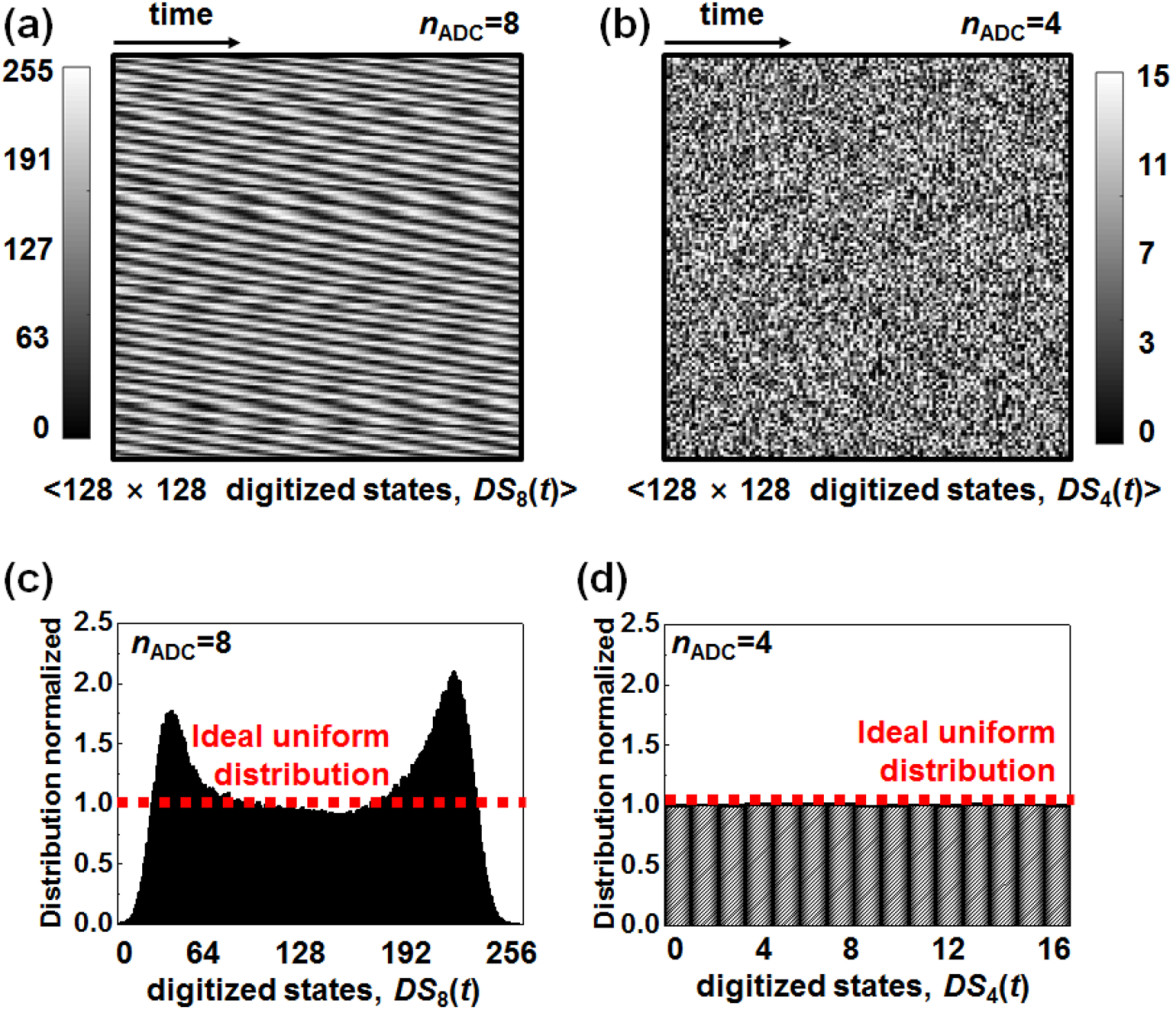
(**a**) Regular patterned contour map with measured digitized states of *DS*_8_(*t*) ranging from 0 to 255 when the number of the extracted ADC bit (*n*_ADC_) is 8. (**b**) Irregular patterned contour map with measured digitized states of *DS*_4_(*t*) ranging from 0 to 15 when *n*_ADC_ = 4. (**c**) Non-uniform probability density distribution of *DS*_8_(*t*) ranging from 0 to 255 when *n*_ADC_ = 8. (**d**) Uniform probability density distribution of digitized states of *DS*_4_(*t*) ranging from 0 to 15 when *n*_ADC_ = 4.

Figure 3a and b visualize the abovementioned regular and irregular properties, respectively, in the form of a 2-dimsensional contour map. Figure 3a exhibits a contour map from the measured *DS*_8_(*t*), while Fig. 3b displays that from the measured *DS*_4_(*t*). These contour maps are expressed in monotoned gray scale where a white and a black color indicate a value of high and low ${DS}_{{n}_{\mathrm{ADC}}}(t)$, respectively. Each dot is filled according to a chronological order of the time evolution. The contour map of the *DS*_8_(*t*) shows a periodic and regular pattern, which was observed in the transient digital states of Fig. 2c. On the contrary, the contour map of the *DS*_4_(*t*) shows an irregular and noisy pattern, which was found in the transient digital states of Fig. 2d. Consequently, the transient digital states of the *DS*_4_(*t*) are more unpredictable than those of the *DS*_8_(*t*) from a graphical point of view. Thus, we can infer that partial adoption of ADC output bits significantly influences the unpredictability and randomness, *i*.*e*., the lower bit such as LSB results in increased unpredictability. Figure S2 displays a contour map of the measured digitized state of the *DS*_8_(*t*) to the *DS*_1_(*t*) according to the number of the employed ADC bits.

Figure 3c and d compare a normalized probability density function of *DS*_8_(*t*) and *DS*_4_(*t*), respectively. Distribution of the original probability density extracted through the ADC hardware is normalized by the number of the digitized states, ${2}^{{n}_{\mathrm{ADC}}}$. For example, *DS*_8_(*t*) is normalized by 2^8^ = 256, whereas *DS*_4_(*t*) is normalized by 2^4^ = 16. Thus, a value of 1 is ideal, which corresponds to perfect uniformity. Closer to 1 implies more unpredictability; thus, it is important to check whether the distribution of the digitized state is uniform or not. The distribution of *DS*_8_(*t*) is non-uniform, while that of *DS*_4_(*t*) is uniform. We conclude that *DS*_4_(*t*) is more unpredictable than *DS*_8_(*t*). Figure S3 displays the normalized distribution of the probability density for other digitized states, *e*.*g*., *DS*_7_(*t*), *DS*_6_(*t*), *DS*_5_(*t*) and *DS*_3_(*t*). As *n*_ADC_ decreases, the distribution of the probability density becomes more uniform, *i*.*e*., more unpredictable.

**Figure 4:**
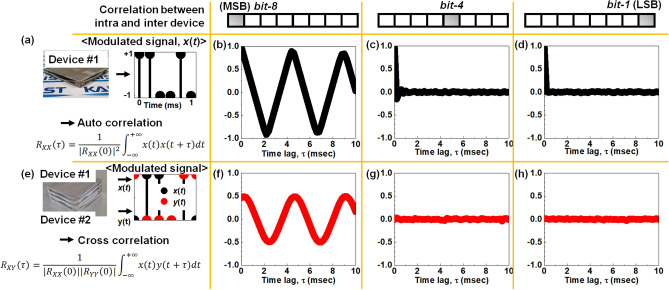
Analyses of predictability in terms of auto correlation for an intra device and cross correlation for inter devices. (**a**) Extraction of auto correlation for an intra device. (**b**) Coefficient of auto correlation for the signal of *bit-8* according to time lag. (**c**) Coefficient of auto correlation for the signal of *bit-4* according to time lag. (**d**) Coefficient of auto correlation for the signal of *bit-1* according to time lag. (**e**) Extraction of cross correlation between inter devices. (**f**) Coefficient of cross correlation for the signal of *bit-8* according to time lag. (**g**) Coefficient of cross correlation for the signal of *bit-4* according to time lag. (**h**) Coefficient of cross correlation for the signal of *bit-1* according to time lag.

Figure 4 shows the auto-correlation and cross-correlation of the extracted digital signals from the ADC digital outputs. The auto-correlation is quantified as a correlation coefficient ranging from − 1 to 1. Closer to 0 indicates less auto-correlation between two values of the same variable but at different times. The cross-correlation is also metrized as another correlation coefficient ranging from − 1 to 1. When the value approaches 0, it indicates that there is less cross-correlation between two variables as their values change at different times relative to each other. The auto-correlation coefficient can measure similarity between variables in an intra device and the cross-correlation coefficient can do the same in inter devices. It is rational to verify randomness and unpredictability by evaluating the auto-correlation and the cross-correlation^31–33^. When a value of the auto-correlation and the cross-correlation coefficient is close to 0, sequences of raw data are independent of each other, randomly distributed, and unpredictable^32^.

The auto-correlation coefficient refers to the self-similarity within a single signal as a function of different delay times^34,35^. The auto-correlation is represented as follows:

$${R}_{XX}(\tau )=\frac{1}{{\left|{R}_{XX}\left(0\right)\right|}^{2}}{\int }_{-\infty }^{+\infty }x\left(t\right)x(t+\tau )dt.$$

All coefficients are normalized by |*R*_*XX*_(0)|^2^, which is the coefficient for no time lag. Thus, the auto-correlation coefficient of *R*_*XX*_(0) is always 1 by definition of the normalized auto-correlation function^35^. A correlation of -1 represents a perfectly negative correlation, while a correlation of 1 indicates a perfectly positive correlation. In contrast, a correlation of 0 refers to no linear relationship between the same variable at different times. This implies that random digital bits are unpredictable for the time evolution^36,37^.

Figure 4a describes the method to extract the auto-correlation coefficient in an intra device. First, digital bits are generated from 8 output pins of the ADC hardware. Second, the sampling process is conducted at every 200 μs. Third, the digital bits are converted to *x*(*t*) with a conversion rule of a logic value: switching of logic ‘0’ to integer ‘− 1’ and switching of logic ‘1’ to integer ‘1’. From these procedures, *x*(*t*) is extracted from the ADC hardware. This conversion makes a fair comparison of the auto-correlation coefficient^38^. Finally, the auto-correlation coefficient of *R*_*XX*_ is extracted from *x*(*t*) for each of digital output. Figure 4b exhibits the auto-correlation coefficient of *bit-8* according to time lag. Its coefficient of the *bit-8* shows periodicity. In other words, the digitized signals of the *bit-8* are predictable owing to the self-similarity for the time evolution. In contrast, the auto-correlation coefficient of *bit-4* and *bit-1* are very rapidly reduced to 0, which infers that there is no relationship with a self-delayed signal, as shown in Fig. 4c and d^36,37^. Figure S4 shows the other auto-correlation coefficients according to the ADC digital output signals from the ADC hardware for an intra device from *bit-8* to *bit-1*.

Figure 4e describes the method to extract the cross-correlation coefficient in inter devices. First, digital bits are generated from two arbitrary devices (e.g., device #1 and device #2) via the ADC hardware. Second, the generated digital bits are converted to discrete-time signals from continuous-time signals by the sampling process. Third, the converted signals are modulated to *x*(*t*) (device #1) and *y*(*t*) (device #2). A modulation rule of a logic value is switching of logic ‘0’ to integer ‘− 1’ and switching of logic ‘1’ to integer ‘1’. Finally, the cross-correlation coefficient of *R*_*XY*_ is extracted from *x*(*t*) and *y*(*t*). The cross-correlation coefficient between inter devices is expressed as follow:

$${R}_{XY}(\tau )=\frac{1}{\left|{R}_{XX}\left(0\right)\right|\left|{R}_{YY}\left(0\right)\right|}{\int }_{-\infty }^{+\infty }x\left(t\right)y(t+\tau )dt.$$

All coefficients are normalized by |*R*_*XX*_(0)||*R*_*YY*_(0)|, which is the multiplication product between two auto-correlation coefficients for no time lag. A correlation of − 1 represents a perfectly negative correlation and a correlation of 1 denotes a perfectly positive correlation. In contrast, a correlation of 0 indicates that there is no linear relationship between the *x*(*t*) and *y*(*t*) variables. Even though *x*(*t*) is known, *y*(*t*) cannot be predicted from the known *x*(*t*) due to the inherent device-to-device randomness^39,40^. Figure 4f exhibits the cross-correlation coefficient of *bit-8* according to time lag. Its coefficient of *bit-8* is periodic, which resembles the auto-correlation coefficient, as shown in Fig. 4b; however, the cross-correlation coefficient of *bit-4* and *bit-1* becomes 0, as shown in Fig. 4g and h. This implies that there is no relationship between the inter-device signals. Figure S5 exhibits the other cross-correlation coefficients from the ADC hardware for inter devices from the *bit-8* to *bit-1* signals. Through analyses of the correlations between intra- and inter-devices, the lower *n*_ADC_ extracted from outputs of the ADC hardware results in increased unpredictability, which is attractive for the improvement of true random number quality.

**Figure 5:**
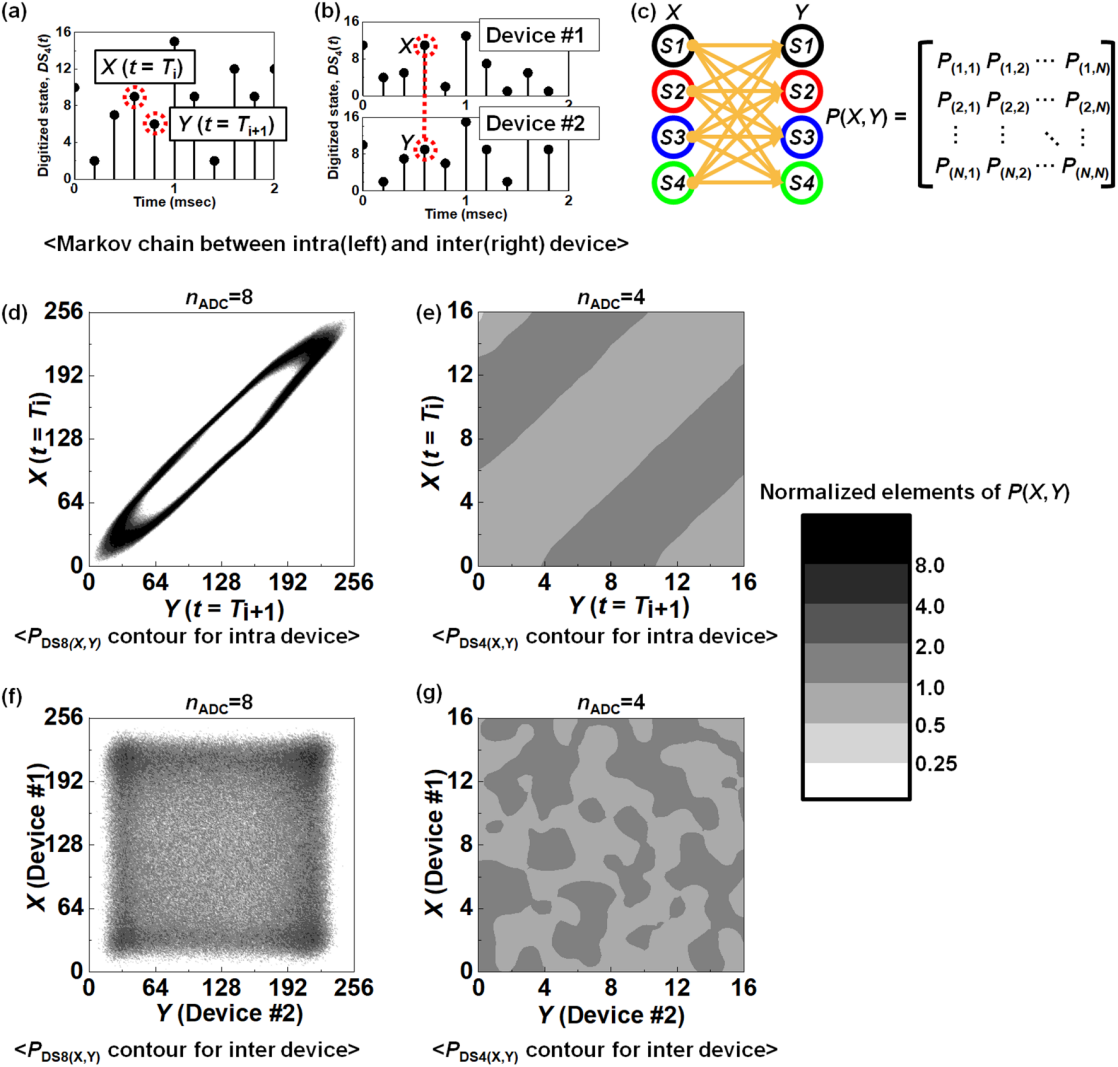
Visualization of the Markov chain model between intra and inter devices. (**a**) Schematic illustration to extract *DS*_*4*_(*t*) in the Markov chain model for an intra device. (**b**) Schematic illustration to extract *DS*_*4*_(*t*) in the Markov chain model between inter devices. The transition probability from *X* state to *Y* state is represented by sub-elements in the *P*(*X*,*Y*) matrix. The Markov chain model for an intra device (left) denotes temporal transition according to the time evolution from the *X* state (*t* = *T*_i_) to *Y* state (*t* = *T*_i+1_), while the Markov chain model between inter devices (right) indicates spatial transition from the *X* state (device #1) to *Y* state (device #2). (**c**) Schematic illustration of the state-transition from the *X* variable to *Y* variable and its matrix form of *P*(*X*,*Y*). (**d**) Contour map of *P*_*DS*8(*X*,*Y*)_ for an intra device according to time evolution. (**e**) Contour map of *P*_*DS*4(*X*,*Y*)_ for an intra device according to time evolution. (**f**) Contour map of *P*_*DS*8(*X*,*Y*)_ among inter devices. (**g**) Contour map of *P*_*DS*4(*X*,*Y*)_ among inter devices.

A Markov chain provides a method to analyze uncertainty and unpredictability through simple matrix calculations^41–43^. In particular, the value of joint entropy and mutual information can be extracted from a Markov chain model, which are mathematical and statistical methods to quantify unpredictability. In Fig. 5, the Markov chain model is visualized for an intra device and inter devices. Their distributions, according to the time evolution, are illustrated in Fig. 5a. Conversely, Fig. 5b describes methods to extract Markov chains for inter devices. Two types of transitions are available. One is a time transition between *X*(*t* = *T*_i_) and *Y*(*t* = *T*_i+1_) for an intra device. *T*_i_ is a time that an ith sampling process is performed, whereas *T*_i+1_ is another time that an (i + 1)^th^ sampling process is conducted. For example, the time transition is investigated between *t* = *T*_i_ and *t* = (*T*_i_ + 200 μs) with a sampling rate of 200 μs, as shown in Fig. 5a. The other is a spatial transition between device #1 and device #2 for the inter devices, as described in Fig. 5b. In other words, the spatial transition is investigated between a digitized state of device #1 and device #2 under an identical time condition, *t* = *T*_i_. As shown in Fig. 5c, the Markov chain model is represented with a probability density function of *P*(*X*,*Y*) as the matrix form. Its elements are *P*_(1,1)_, *P*_(1,2)_, ··· , and *P*_(*N*,*N*)_. The probability density function of *P*(*X*,*Y*) is also called the joint probability distribution regarding two random variables *X* and *Y*. *N* is the number of the state. When the number of the extracted ADC bit is *n*_ADC_, *N* = ${2}^{{n}_{\mathrm{ADC}}}$. Thus, when the number of the extracted ADC bit is *n*_ADC_, the number of *P*(*X*,*Y*) matrix elements is *N*^2^ ($={2}^{{n}_{\mathrm{ADC}}}\times {2}^{{n}_{\mathrm{ADC}}})$. In detail, *P*(*X*,*Y*) is the probability density function when an *X* state is transited to a *Y* state. The *X* state and *Y* state are discrete random variables, which represent a whole set of feasible values during the experiments^44,45^. *X* is a random variable before the state transition occurs, while *Y* is also another random variable after the state transition. For example, random variable *X* = {*S1*, *S2*, *S3*, *S4*} and *Y* = {*S1*, *S2*, *S3*, *S4*} for *n*_ADC_ = 2. In this case, the number of the state is 4 and the number of the elements in the *P*(*X*,*Y*) matrix is 16 ($={2}^{{n}_{\mathrm{ADC}}}\times {2}^{{n}_{\mathrm{ADC}}})$, *i*.*e*., the *P*(*X*,*Y*) matrix is represented with {*P*_(1,1)_, *P*_(1,2)_, *P*_(1,3)_,‧‧‧, *P*_(4,2)_, *P*_(4,3)_, *P*_(4,4)_}. Figure 5c graphically illustrates the state transition from *X* to *Y* and mathematically shows its corresponding matrix form. For example, *P*_(4,2)_ indicates the probability density that state-4 (abbreviated to *S4*) in the random variable *X* is transited to state-2 (abbreviated to *S2*) in the random variable *Y*. The probability density is normalized so that the total summation of all the *P*(*X*,*Y*) matrix elements is in unity. As another example, the *P*(*X*,*Y*) matrix can have 256 (= 2^4^ × 2^4^) elements for *n*_ADC_ = 4. Therefore, Fig. 5a and b represent the transition of *DS*_4_(*t*) from random variable *X* = {*S1*, *S2*, ‧‧‧, *S15*, *S16*} to random variable *Y* = {*S1*, *S2*, ‧‧‧, *S15*, *S16*}.

Figure 5d shows a contour map of the elements from the Markov chain model for an intra device of *DS*_8_(*t*) and Fig. 5e exhibits that of *DS*_4_(*t*). The Markov chain model for an intra device computes the state transition according to the time evolution from the *X*-state (*t* = *T*_i_) to the *Y*-state (*t* = *T*_i+1_). All the elements were previously normalized and a value of 1 is ideal. This implies perfect uniformity, which is highly desirable for unpredictability. The contour map of Fig. 5d depicts the non-uniform distribution of the digital bits. In non-uniform distribution, a next state is predictable if a current state is revealed. In contrast, the contour map of Fig. 5e shows a uniform distribution of the digital bits ranging from 0.5 to 2 in a greyscale bar. Therefore, a uniform distribution of the digital bits, as in the 4 bits case, is preferred for unpredictability. Figure S6 displays the contour map for *DS*_8_(*t*), *DS*_7_(*t*), *DS*_6_(*t*), *DS*_5_(*t*), *DS*_4_(*t*), and *DS*_3_(*t*). The Markov chain model for an intra device shows that unpredictability is enhanced as the number of bits is reduced.

Figure 5f exhibits the contour map of the Markov chain model for inter devices of *DS*_8_(*t*), while Fig. 5g shows that of *DS*_4_(*t*). In terms of RNG operations, it is critically important to generate unpredictable signals for the identical input to different devices^46,47^. The Markov chain model between inter devices computes the state transition between different devices from the *X*-state (device #1) to the *Y*-state (device #2). All the elements were previously normalized and a value of 1 is ideal. The contour map of Fig. 5e exhibits non-uniform distribution where the normalized *P*(*X*,*Y*) at certain regions is larger than 2. As discussed above, if a state of one device is revealed, that of the other devices is predictable for the non-uniform distribution. In contrast, the contour map of Fig. 5g displays uniform distribution and the normalized *P*(*X*,*Y*) is ranges from 0.5 to 2. Consequently, the digital bits from the lower *n*_ADC_ are preferred for the unpredictability in the Markov chain model. Figure S7 shows the contour map between the inter devices for *DS*_8_(*t*), *DS*_7_(*t*), *DS*_6_(*t*), *DS*_5_(*t*), *DS*_4_(*t*), and *DS*_3_(*t*).

It is also important to evaluate the unpredictability by a mathematical and statistical method, *e.g.*, joint entropy and mutual information^48–51^. The evaluation of the unpredictability in terms of joint entropy and mutual information is classified into two cases: analyses with an intra device and inter devices, according to the number of extracted bits from the ADC. Thus, four unpredictability metrics are employed by the mathematical and statistical method. Case I is the joint entropy with the intra device, Case II is the joint entropy with inter devices, Case III is mutual information with the intra device, and Case IV is mutual information with inter devices. For a given joint probability distribution *P*(*X*,*Y*) between a random variable *X* and *Y*, *P*(*X*,*Y*) is a measure of the uncertainty associated with a set of variables by checking *P*(*X*,*Y*) = *P*(*X*)·*P*(*Y*)^52,53^. Probability distributions of *P*(*X*,*Y*), *P*(*X*), and *P*(*Y*) are represented as follows.

$$\begin{aligned} & P\left( {X, Y} \right) = \left[ {\begin{array}{*{20}c} {P_{{\left( {1,1} \right)}} } & \cdots & {P_{{\left( {1,N} \right)}} } \\ \vdots & \ddots & \vdots \\ {P_{{\left( {N,1} \right)}} } & \cdots & {P_{{\left( {N,N} \right)}} } \\ \end{array} } \right] \\ & P\left( X \right) = \left[ {\begin{array}{*{20}c} {P_{1} } & { \cdots P_{j} \cdots } & {P_{N} } \\ \end{array} } \right], \;\;{\text{where}} P_{j} = \mathop \sum \limits_{l = 1}^{N} P_{{\left( {j,l} \right)}} \\ & P\left( Y \right) = \left[ {\begin{array}{*{20}c} {P_{1} } & { \cdots P_{k} \cdots } & {P_{N} } \\ \end{array} } \right], \;\;{\text{where}} P_{k} = \mathop \sum \limits_{m = 1}^{N} P_{{\left( {k,m} \right)}} \\ \end{aligned}$$

The summation for all the components is 1, *i*.*e*.,

$$\sum_{k=1}^{N}\sum_{j=1}^{N}{P}_{(j,k)}=1.$$

*P*(*X*,*Y*) is the joint probability distribution between a random variable *X* and *Y*. Figure 5d shows the contour map for the intra device when *n*_ADC_ = 8, Fig. 5e exhibits that for the intra device when *n*_ADC_ = 4, Fig. 5f displays that for inter devices when *n*_ADC_ = 8, and Fig. 5g plots that for inter devices when *n*_ADC_ = 4.

In contrast to the joint probability *P*(*X*,*Y*), *H*(*X*,*Y*) is joint entropy, which is a metric that shows the quantity correlation between random variables *X* and *Y*^54,55^. The joint entropy is represented with the joint probability distribution, *P*(*X*,*Y*), as follows.

$$H\left(X, Y\right)\equiv -\sum_{j=1}^{N}\sum_{k=1}^{N}{P}_{(j,k)}\cdot {\mathrm{log}}_{2}{P}_{(j,k)}$$

The joint entropy measures the uncertainty by checking *P*(*X*,*Y*) = *P*(*X*)·*P*(*Y*), as mentioned above^52,53^. A high level of joint entropy implies that the random variable *Y* is unpredictable even though the random variable *X* is unwantedly known. For Case I (joint entropy with the intra device), *H*(*X*(*t*),*Y*(*t*)) reflects how unpredictable a state transition between different times is. The different times indicate a time lag between *X*(*t* = *T*_i_) and *Y*(*t* = *T*_i+1_). For Case II (joint entropy with inter devices), *H* (*X*(*t*),*Y*(*t*)) represents the spatial correlation strength between random variables *X* (device #1) and *Y* (deice #2), *i*.*e*., it quantifies a level of the spatial correlation.

Mutual information *I*(*X*,*Y*) is another metric to describe the uncertainty. This is simply calculated by the following.

$$\begin{aligned} & I\left( {X,{ }Y} \right) \equiv H\left( X \right) + H\left( Y \right) - H\left( {X,Y} \right) \\ & H\left( X \right) = - \mathop \sum \limits_{j = 1}^{{2^{n} }} P_{j} \cdot \log_{2} P_{j} ,{ }\;\;{\text{where }}P_{j} = \mathop \sum \limits_{l = 1}^{N} P_{{\left( {j,l} \right)}} \\ & H\left( Y \right) = - \mathop \sum \limits_{k = 1}^{{2^{n} }} P_{k} \cdot \log_{2} P_{k} ,{ }\;\;{\text{where }}P_{k} = \mathop \sum \limits_{m = 1}^{N} P_{{\left( {j,m} \right)}} \\ \end{aligned}$$

Smaller *I*(*X*,*Y*) refers to when the state transition becomes more unpredictable^56,57^. For Case III (mutual information with the intra device), mutual information with temporal variation quantifies a level of time correlation between time intervals by tracing information with past observations at the same position. Therefore, an ideal value of *I*(*X*,*Y*) = 0 represents that the observation between infinitely long time intervals cannot influence the unpredictability^58,59^. For Case IV (mutual information with inter devices), mutual information with spatial variation quantifies a level of device-to-device correlation at the identical time condition. Thus, an ideal value of *I*(*X*,*Y*) = 0 denotes that the generated digitized numbers of device #1 are perfectly unpredictable, although the digitized numbers of device #2 are totally revealed.

**Figure 6:**
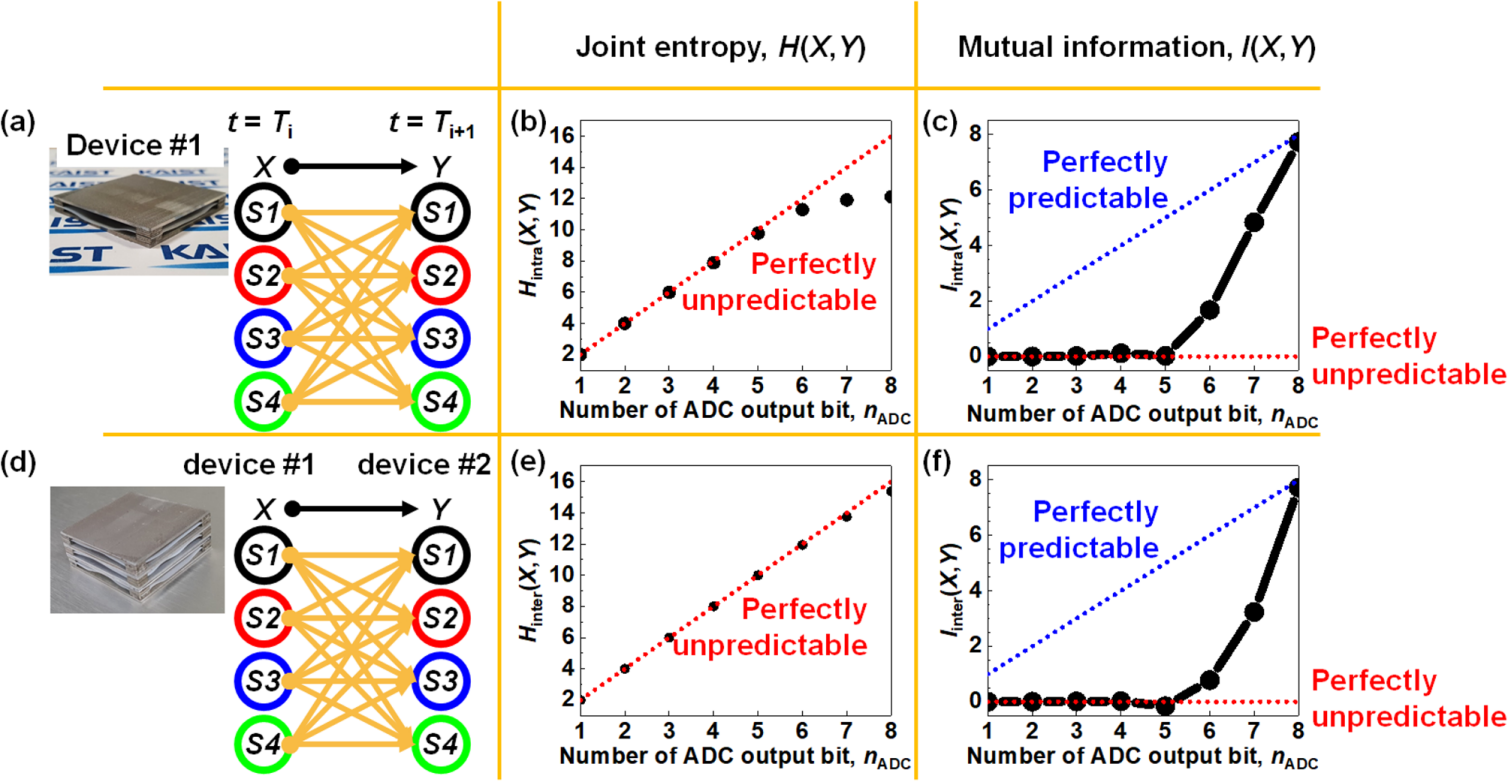
Evaluation of unpredictability in terms of joint entropy *H*(*X*,*Y*) and mutual information *I*(*X*,*Y*) for both an intra device and inter devices according to the number of the ADC output bit (*n*_ADC_). (**a**) Extraction of *H*_intra_(*X*,*Y*) and *I*_intra_(*X*,*Y*) in a single device at the same input. (**b**) Extracted *H*_intra_(*X*,*Y*) between different times. (**c**) Extracted *I*_intra_(*X*,*Y*) between different times. (**d**) Extraction of *H*_inter_(*X*,*Y*) and *I*_inter_(*X*,*Y*) between inter devices at the same time. (**e**) Extracted *H*_inter_(*X*,*Y*) between device #1 and device #2. (**f**) Extracted *I*_inter_(*X*,*Y*) between device #1 and device #2.

Figure 6a describes the method to extract joint entropy *H*_intra_(*X*,*Y*) and mutual information *I*_intra_(*X*,*Y*) between time intervals at the same device. The *X* is a variable for a current state (*t* = *T*_i_) and the *Y* is another variable for a later state (*t* = *T*_i+1_). Figure 6b and c exhibit *H*_intra_(*X*,*Y*) and *I*_intra_(*X*,*Y*) extracted from a single device, respectively. The *H*_intra_(*X*,*Y*) shows the unpredictability when *n*_ADC_ is less than 6. In contrast, *I*_intra_(*X*,*Y*) shows the unpredictability when *n*_ADC_ is less than 5. As a result, the unpredictability of the proposed W-RNG is robust to time evolution when *n*_ADC_ is less than 5, *i*.*e*., *n*_ADC_ ≤ 5.

On the other hand, Fig. 6d describes a method to extract joint entropy *H*_inter_(*X*,*Y*) and mutual information *I*_inter_(*X*,*Y*) between inter devices at the same time. The *X* is a variable for a state in device #1 and the *Y* is a variable for another state in device #2. Figure 6e and f plot *H*_inter_(*X*,*Y*) and *I*_inter_(*X*,*Y*) extracted from different devices, respectively. These show the device-to-device unpredictability with an identical input condition. The *H*_inter_(*X*,*Y*) approves that the unpredictability is guaranteed for all *n*_ADC_. On the contrary, *I*_inter_(*X*,*Y*) supports that the unpredictability is available when *n*_ADC_ is less than 5. As a result, the unpredictability of the proposed W-RNG is robust to device-to-device variation when *n*_ADC_ is less than 5. For visualization, Fig. S8 illustrates the relationship between *H*(*X*,*Y*) and *I*(*X*,*Y*) in Venn diagram form.

**Figure 7:**
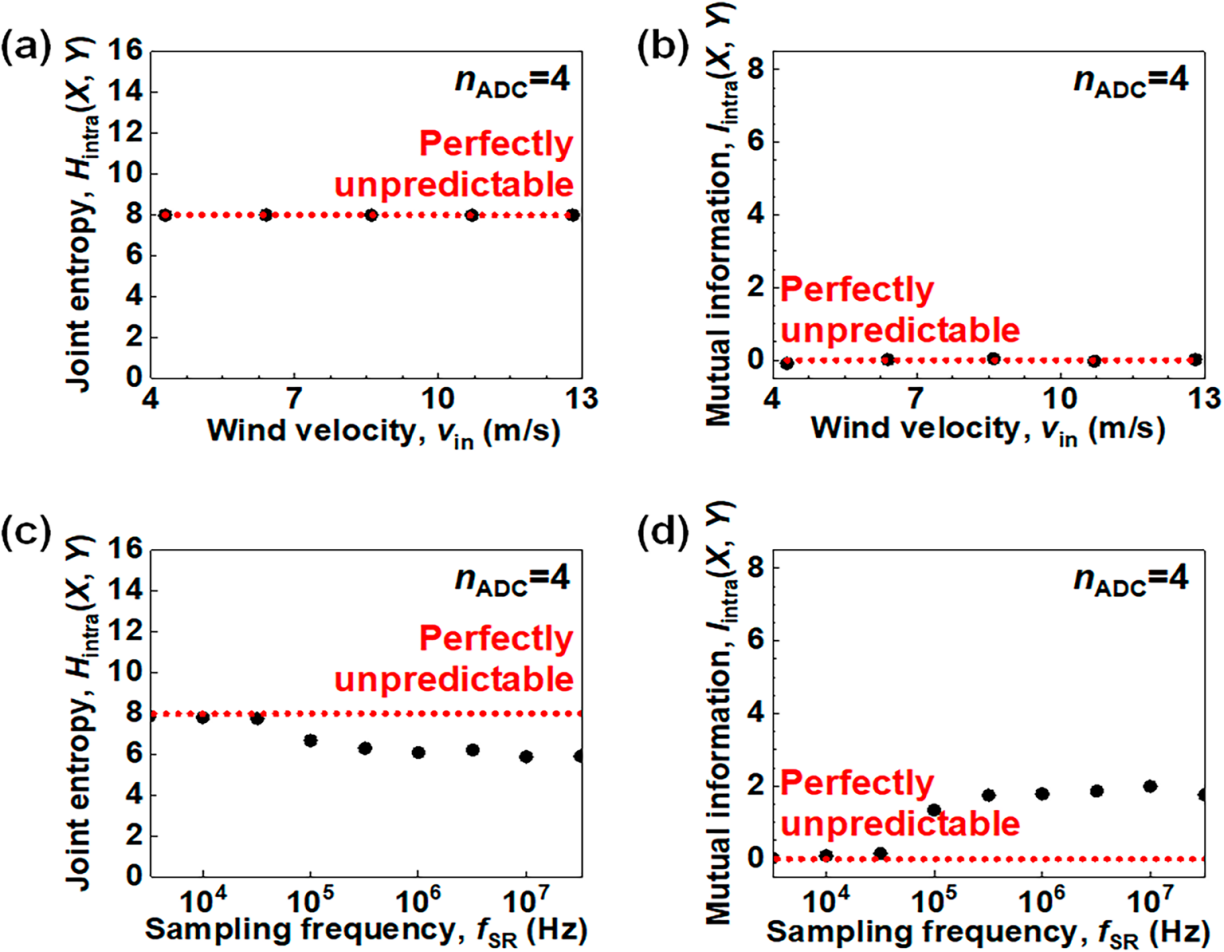
Evaluation of unpredictability according to wind velocity (*v*_in_) and sampling frequency (*f*_SR_) for an intra device when the number of the ADC output bit (*n*_ADC_) is 4. (**a**) *H*_intra_(*X*,*Y*) vs. *v*_in_. (**b**) *I*_intra_(*X*,*Y*) vs. *v*_in_. (**c**) *H*_intra_(*X*,*Y*) vs. *f*_SR_. (**d**) *I*_intra_(*X*,*Y*) vs. *f*_SR_.

Figure 7 shows the unpredictability according to wind velocity (*v*_in_) introduced to an air gap (*h*) of the W-RNG, and the sampling rate frequency (*f*_SR_) for *n*_ADC_ = 4. The *H*_intra_(*X*,*Y*) and *I*_intra_(*X*,*Y*) are used as a metric to evaluate the unpredictable property for the time evolution. The red dotted lines in Fig. 7 indicate ideal unpredictable signals. Figure 7a and b exhibit *H*_intra_(*X*,*Y*) and *I*_intra_(*X*,*Y*) for various *v*_in_ ranging from 4.2 m/s to 12.8 m/s, respectively. They are also perfectly unpredictable regardless of *v*_in_. Figure 7c and d display *H*_intra_(*X*,*Y*) and *I*_intra_(*X*,*Y*) for various *f*_SR_ ranging from 10 kHz to 30 MHz. It is well known that the throughput of an RNG linearly increases when an *f*_SR_ becomes larger. However, Fig. 7c and d show that unpredictability is adversely reduced as the *f*_SR_ is over 100 kHz. Consequently, unpredictability is sustained as long as the *v*_in_ is over 4.2 m/s, corresponding to a gentle breeze wind, and the *f*_SR_ is below 100 kHz.

**Figure 8:**
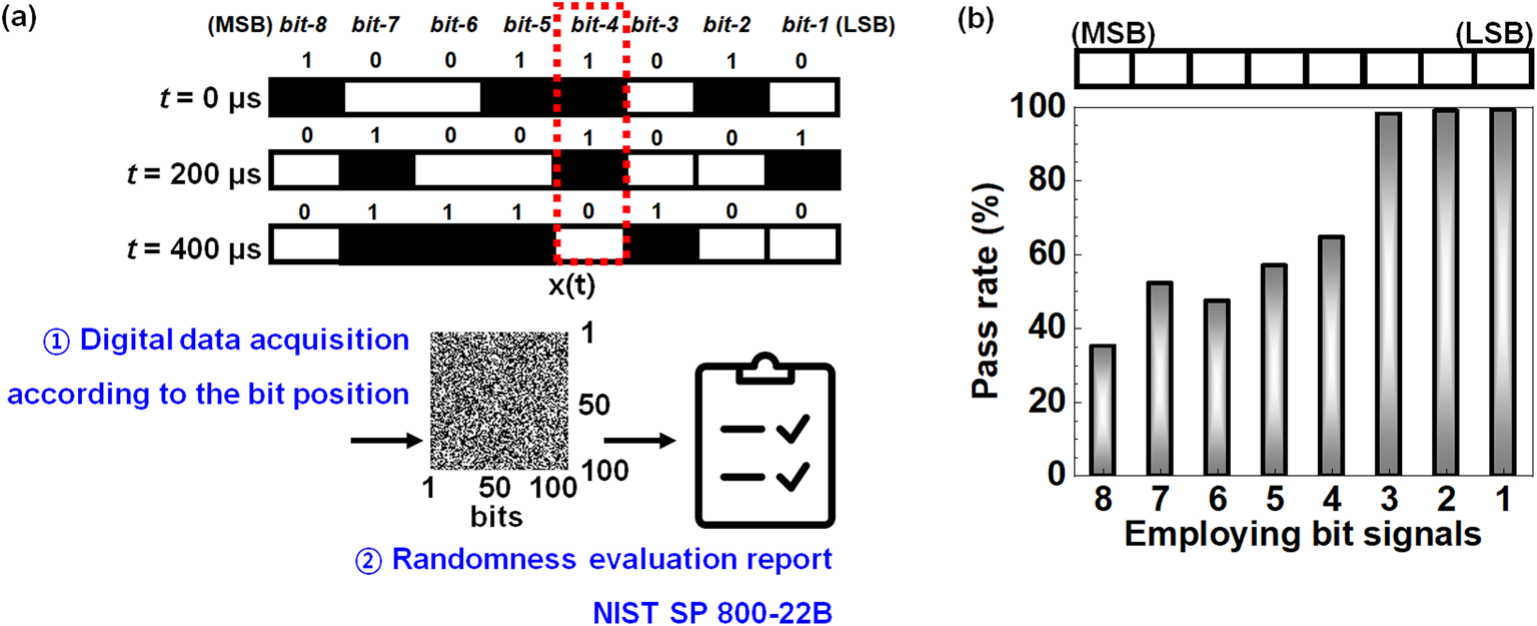
(**a**) Schematic of procedure to evaluate the NIST SP 800-22B test according to the ADC digital output signals from *bit-8* (MSB) to *bit-1* (LSB). (**b**) Average pass rate for all sub-suites of NIST SP 800-22B according to the ADC digital output signals from *bit-8* (MSB) to *bit-1* (LSB).

To evaluate the randomness for each ADC digital output signal, a test suit of the NIST SP 800-22B, which is an authoritative standard to evaluate the randomness of signals generated from an RNG, was used. Figure 8a shows the procedure of making a randomness evaluating report via digital data acquisition. First, digital data of the voltage is generated from the output of 8-pins of the ADC hardware. Next, a randomness test is performed for each ADC digital output signal. Figure 8b represents an average pass rate for the signals of *bit-8* to *bit-1*. This indicates the average value of the pass rate for 15 sub-suites in the NIST SP 800-22B: (1) frequency (mono-bit), (2) frequency within a block, (3) runs, (4) longest run of ones, (5) binary matrix rank, (6) discrete Fourier transform, (7) non-overlapping template matching, (8) overlapping template matching, (9) Maurer’s universal statistical, (10) linear complexity, (11) serial, (12) approximate entropy, (13) cumulative sums, (14) random excursions, and (15) random excursions variant. These 15 sub-suites are simply classified by four groups according to bit capacities, which are 10 kbits, 1 Mbits, 38,912 bits and 65,536 bits. Sub-suits (1), (2), (3), (4), (11), (12) and (13) tests were conducted 400 times with 10 kbits^60,61^. Sub-suits (7), (8), (9), (10), (14), and (15) tests were conducted 4 times with 1 Mbits. A sub-suit (5) binary matrix rank test was performed 102 times with 38,912 bits. The other sub-suite of (6) discrete Fourier transform was performed 61 times with 65,536 bits. For all the experiments, a significance level (*α*) was set to 0.01. The NIST recommends that *α* be set to a range between 0.001 and 0.01^61^. If the *p*-value for a sequence test is greater than or equal to *α*, it is considered to have passed the test. In conclusion, digital signals of *bit-3* to *bit-1* possess excellent random properties, whereas those of *bit-8* to *bit-4* are worsened. This data reveals that the random properties are influenced by the bit-*n* ranged from 8 (MSB) to 1 (LSB) and the reduced randomness results in degraded unpredictability.

## Conclusion

This study demonstrated the use of a wind-driven triboelectric nanogenerator, named W-RNG, as a dual-function device that can harvest energy and generate random numbers. The W-RNG has a two-in-one structure. The partial adoption of output digital signals from analog-to-digital converter (ADC) hardware significantly enhanced unpredictable properties, which were analyzed by statistical and mathematical metrics such as auto-correlation, cross-correlation, joint entropy, and mutual information. Auto-correlation and cross-correlation analyses between intra and inter devices reveal that digital signals of *bit-4* to *bit-1* possessed more unpredictability compared with those of *bit-8* to *bit-5*. On the other hand, digital signals showed unpredictable properties in terms of joint entropy and mutual information when the number of ADC bit (*n*_ADC_) is less than 5. Therefore, the data adoption of *bit-4* among the raw digital bits guaranteed unpredictable properties. These results can pave way to quickly checking whether a used entropy source for a true random number generator (TRNG) is indeed unpredictable in both a theoretical and statistical point of view.

### Supplementary Information

Supplementary Information.

## Data Availability

The datasets generated during and/or analysed during the current study are available from the corresponding author on reasonable request.
