# Supplementary material for: Analyses of unpredictable properties of a wind-driven triboelectric random number generator
Source: Sci Rep. 2023 Oct 3;13:16610. doi: 10.1038/s41598-023-43894-1 (PMC10547768; doi:10.1038/s41598-023-43894-1)
Supplement: Supplementary file 1 — Supplementary Information. [file 41598_2023_43894_MOESM1_ESM.docx]

**Supplementary Information**

**Analyses of unpredictable properties of a wind-driven triboelectric random number generator**

Moon-Seok Kim,^1,2^ Il-Woong Tcho,^1^ and Yang-Kyu Choi^1^

^1^ School of Electrical Engineering, Korea Advanced Institute of Science and Technology (KAIST), 291 Daehak-ro, Yuseong-gu, Daejeon 34141, Republic of Korea

*^2^Department of Semiconductor System Engineering, Hanbat National University, 125 Dongseo-daero, Yuseong-gu, Daejeon 31538, Republic of Korea*

*Authors to whom correspondence should be addressed.

Email address: [ykchoi@ee.kaist.ac.kr](mailto:ykchoi@ee.kaist.ac.kr)

**1.** **Power generation from W-TENG**

Figure S1 depicts power generation from a wind-driven triboelectric nanogenerator (W-TENG) mounted on a commercial cross-shaped drone. Wind pressure arising from a rotary wing of the drone acts as the energy source to the W-TENG, which converts wind energy to electrical energy. Figure S1a shows the mounted W-TENG with a light emitting diode (LED) on the drone. Figure S1b illustrates the electrical configuration of the W-TENG and the LED. The LED is used to visually confirm power generation by the W-TENG. The center region of the drone feels the highest wind pressure because it can collect wind energy from the 4 rotary wings located at each end of the arm in the cross-shaped drone [1-2]. Figure S1c exhibits images of the turned-on and turned-off LED controlled by the power from the W-TENG. The photographs show that W-TENG is able to generate power by virtue of the drone’s operation. To clearly distinguish whether the LED is turned on or not, raw images are transformed to gray-scaled images and color-inverted images. The color inversion indicates that each amplitude to a red, green, and blue pixel is reversed, respectively. For example, a blue color represented with (0, 0, 255) in the formant of (red, green, blue) is transformed to another color expressed with (255, 255, 0) corresponding to yellow by the color inversion.


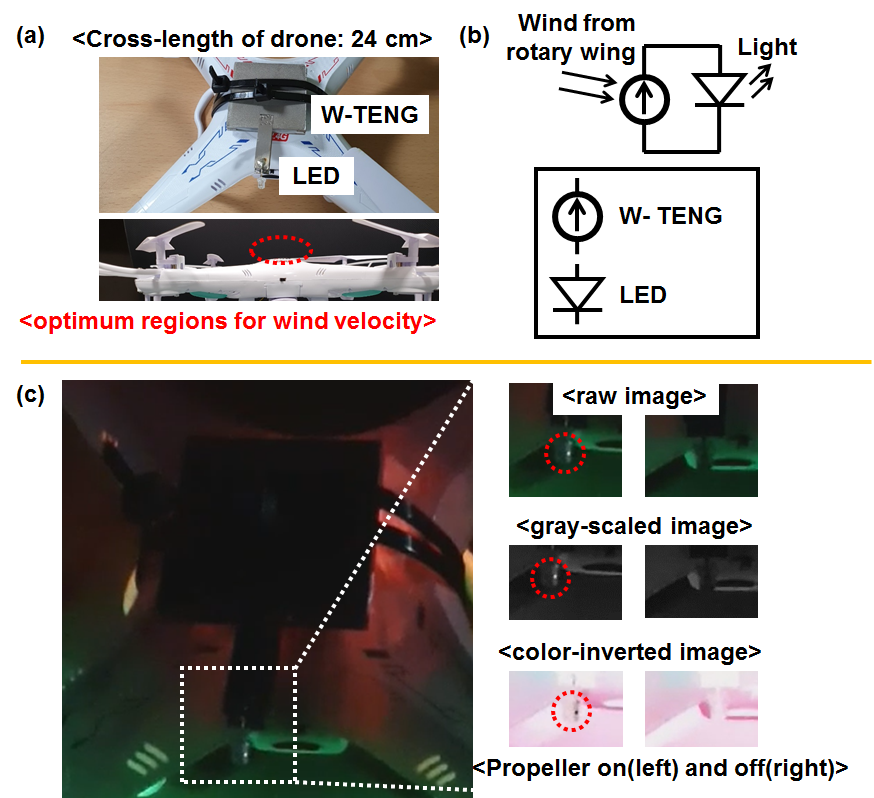


**Figure S1.** (a) Manufactured W-TENG connected with an LED mounted on the drone. (b) Circuit diagram between the W-TENG and the LED. (c) Optical photographs of the LED in a turned-on and turned-off state. Raw images are transformed to gray-scaled image and color-inverted image to clearly distinguish the turned-on and turned-off state.

**2. Comparison of power consumption in analog-to-digital converter**

Table S1 lists the comparison of power consumption in various analog-to-digital converter (ADC) with different sampling frequency and bit resolution. For a self-powered system, generated output power from the W-TENG should be larger than consumed power by an ADC connected to the TRNG (W-TENG). It is worth noting that output power from the W-TENG is 0.79 mW, whereas power consumption of an ADC with the small number of bits is below 200 nW according to the previous researches.

Power consumption of an ADC is dominated by a couple of parameters such as sampling frequency and the number of bits (bit resolution). There is the tendency that power consumption of an ADC is rapidly increased as the number of bits is increased. Thus, we adopt energy-efficient ADC whose bit resolution is 8 bit. Following table compares power consumption of an ADC according to sampling frequency and the number of bits.

**Table S1**. Comparison table of power consumption for various system-on-chip (SoC) based ADCs with different sampling frequencies and bit resolutions.

| **Previous works** | **Sampling frequency** | **Bit resolution** | **Power consumption** |
| --- | --- | --- | --- |
| S. Chang *et al.,* [3] | 3 kHz | 8 | 87 nW |
| Y. Yang *et al.,* [4] | 20 kHz | 8 | 151 nW |
| M. Yip *et al.,* [5] | 5 kHz | 8 | 146 nW |
| G. V. Deepthi *et al.,* [6] | 20 kHz | 32 | 589 μW |
| P. Butler [7] | 1 MHz | 16 | 105 mW |

**3. Contour map of digital bits from analog-to-digital converter**

Figure S2 displays a contour map of the measured digitized state according to the number of bits (*n*_ADC_), which were initially analog bits generated from the W-RNG then finally transformed to digital bits by the ADC hardware. The *n*_ADC_ ranges from 8 (MSB) to 1 (LSB). The digitized state is represented with *DS*_8_(*t*) to *DS*_1_(*t*) according to the *n*_ADC_. Each contour map for the *DS*_8_(*t*) to *DS*_1_(*t*) is plotted in Fig. S2a to Fig. S2h, respectively. Each pixel in the contour map is displayed in chronological order, *i*.*e*., a position moves rightwardly as time elapses. In the contour maps, a value in the *y*-axis corresponds to an *n*_ADC_^th^ state, which ranges from 0, 1, …, $2^{n_{\mathrm{ADC}}}-1$ and indicated in a gray scale. The darker black indicates a lower state. As the *n*_ADC_ decreases, the contour map becomes more irregular. For example, the *DS*_8_(*t*) in Fig. S2a exhibits a periodic pattern; however, the *DS*_4_(*t*) in Fig. S2e shows an irregular pattern. Therefore, a lower *n*_ADC_ results in increased unpredictability of the random numbers.


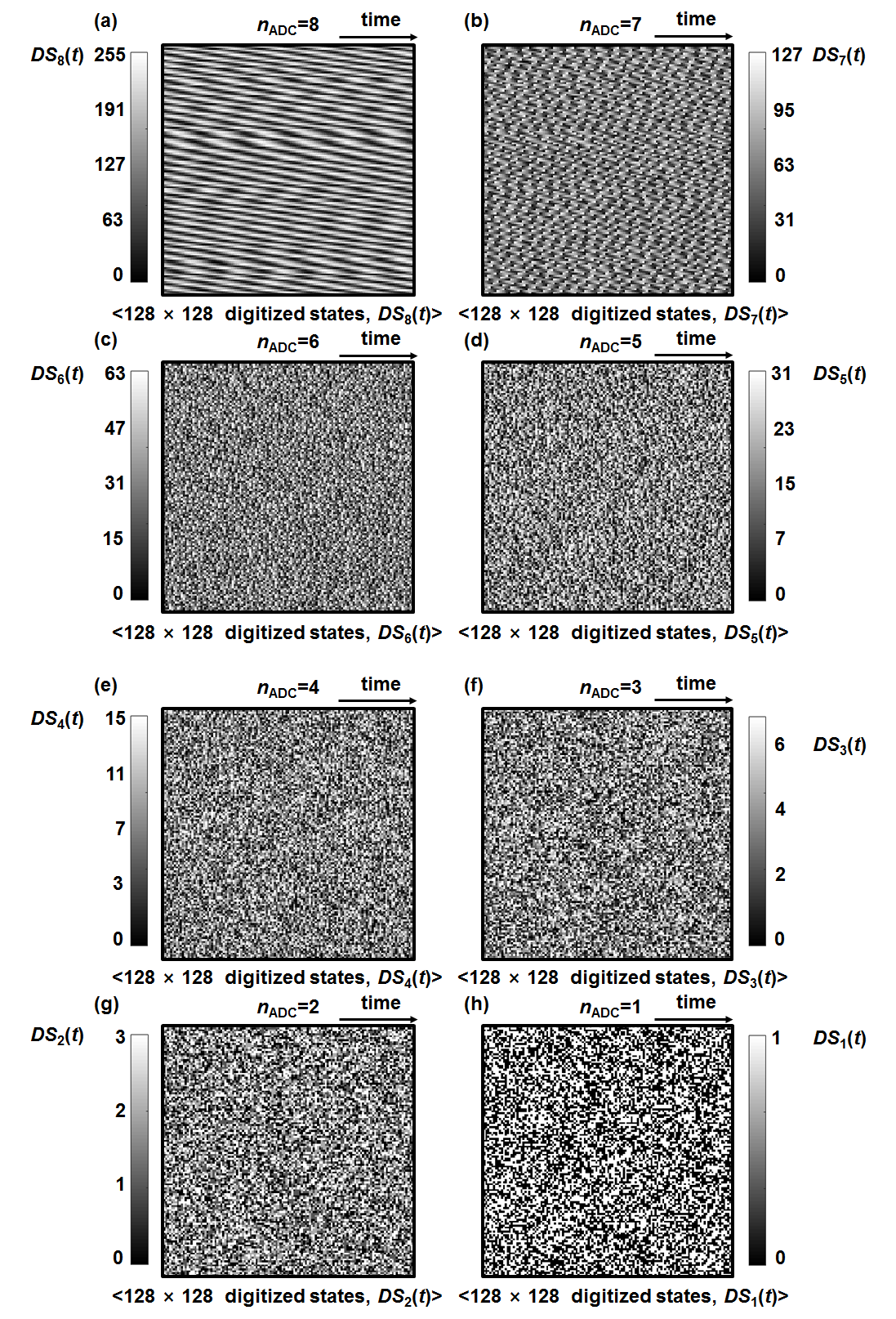


**Figure S2.** Contour map of the measured digitized state *via* the ADC from the W-RNG according to *n*_ADC_: (a) 8, (b) 7, (c) 6, (d) 5, (e) 4, (f) 3, (g) 2, and (h) 1.

**4. Distribution of digitized states**

Figure S3 shows the distribution of the digitized states (${DS}_{n_{\mathrm{ADC}}}(t)$) according to the *n*_ADC_. It was normalized to be 1, which is an ideal value for the perfect uniformity, *i*.*e*., each density of 1 to $2^{n_{\mathrm{ADC}}}-1$ is equally distributed.

As the *n*_ADC_ is reduced, the distribution is flattened to be 1. When the *n*_ADC_ is smaller than 4 in Fig. S3e and Fig. S3f, the ${DS}_{n_{\mathrm{ADC}}}(t)$ becomes flat and uniform. This ideal uniformity is necessary to realize unpredictability. In other words, the non-uniform distribution shown in Fig. S3a, Fig. S3b, and Fig. S3c can be predictable.

**
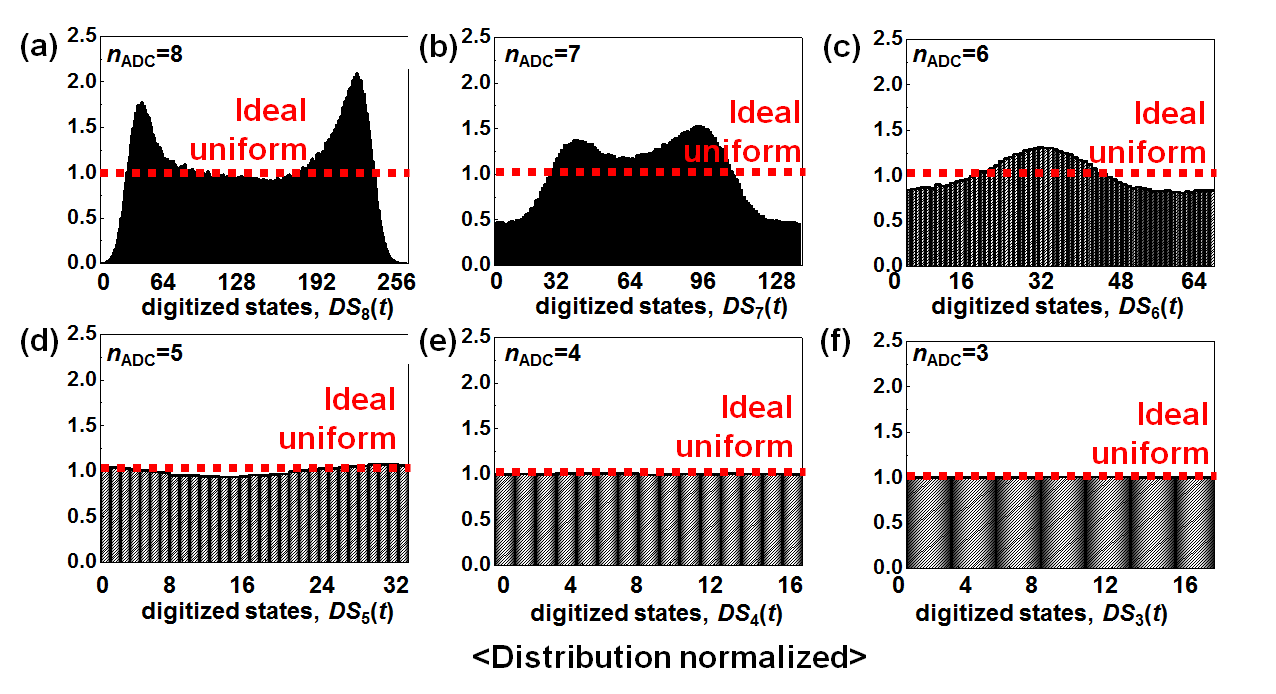
**

**Figure S3.** Distribution of digitized states (${DS}_{n_{\mathrm{ADC}}}(t)$) according to the *n*_ADC_: (a) 8, (b) 7, (c) 6, (d) 5, (e) 4, and (f) 3.

**5. Analysis of auto-correlation for an intra device**

Figure S4 shows the auto-correlation coefficient *via* 8 output pins of the ADC hardware. The coefficient is extracted for an intra-device according to the time evolution. Analysis of the auto-correlation is an alternative approach to verify randomness and unpredictability for an intra device [8-9]. Because an ideal auto-correlation coefficient for perfect randomness is 1, a lower value of the auto-correlation coefficient indicates that the sequence of generated signals becomes independent of each other and less predictable [10-11]. Additionally, the extracted auto correlation from a single device refers to the temporal correlation with the identical signal according to a time lag for an intra device. In other words, the auto-correlation function refers to the self-similarity of the signal over different delay times. The correlation of a signal with a delayed copy of itself as a function of delay is extracted as follows.

$$R_{XX}(\tau)=\frac{1}{\left| R_{XX}\left( 0 \right) \right|^{2}}\int_{-\infty}^{+\infty} x\left( t \right)x(t+\tau)dt$$

All the coefficients are normalized by |*R_XX_*(0)|^2^, which is the coefficient when the time lag is zero. Thus, the auto-correlation coefficient of the *R_XX_*(0) is always 1 according to the definition of the normalized auto-correlation function. *R_XX_*(*τ*) ranges between -1 and 1. Herein, -1 is the perfect negative correlation and 1 is the perfect positive correlation. In contrast, the correlation of 0 implies that two variables are independent of each other, *i*.*e*., even though one variable is known, the other one is untraceable [10-11]. The procedure to extract the auto-correlation coefficient of *R_XX_*(*τ*) is described as follows. First, digital data is generated from a single device *via* the output of 8-pins of the ADC hardware in the form of voltage. Second, digital bits are converted to -1 or 1 by use of the switching rule: a logic value of ‘0’ is assigned as -1 and that of ‘1’ is assigned as 1. This conversion makes fair comparison feasible among the auto-correlation coefficients for various *n*_ADC_ [12]. Last, the *R_XX_* is extracted as a function of the time evolution. Figure S4a to S4h plots the *R_XX_* according to the *n*_ADC_ from 8 to 1, respectively. For example, Fig. S4a shows the *R_XX_* for a *bit-8* (MSB) and Fig. S4h exhibits the *R_XX_* for a *bit-1* (LSB) signal.

The *R_XX_* of the *bit-8* signal is periodic and scarcely decayed, *i*.*e*., it has self-similarity; thus, it is predictable. On the contrary, the *R_XX_* of the *bit-4* signal rapidly converges to 0, *i*.*e*., it does not have self-similarity, predictability and any relationship between self-delayed signals.


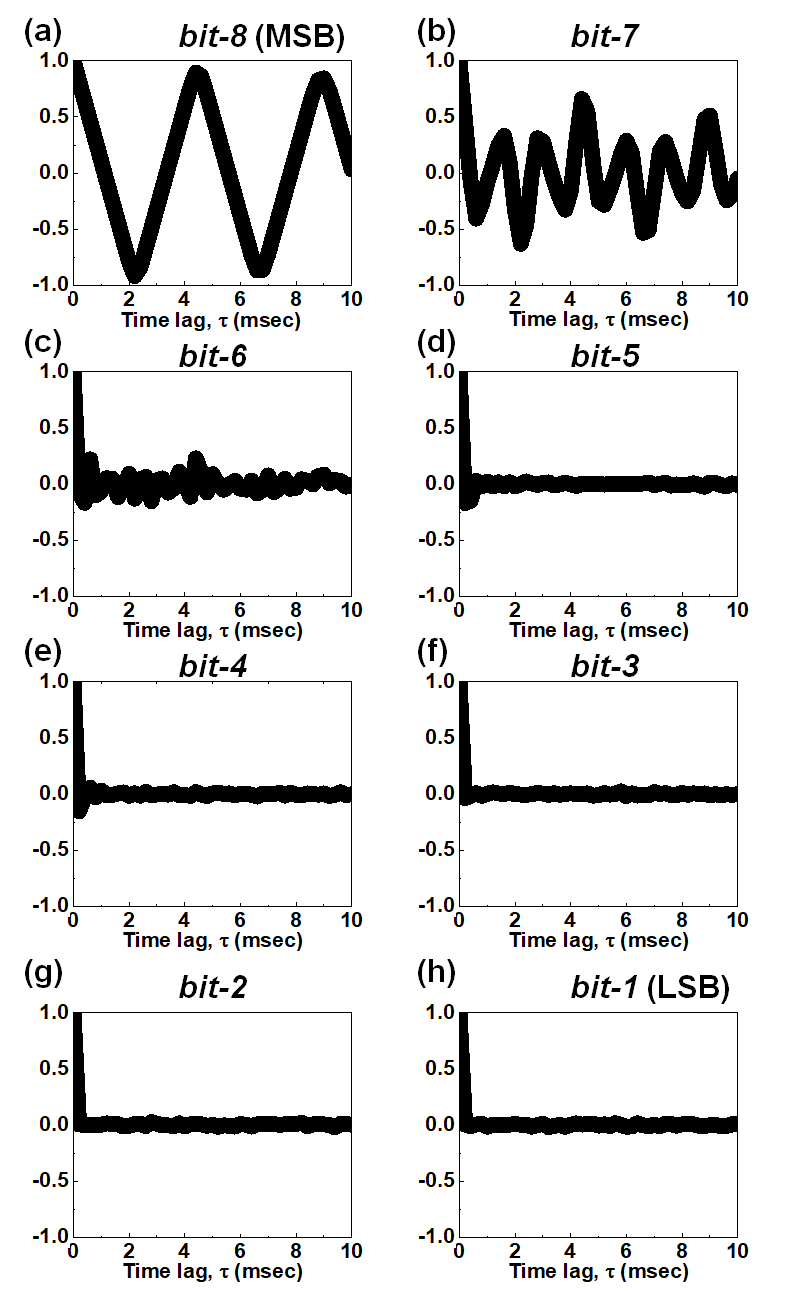


**Figure S4.** Auto-correlation coefficient (*R_XX_*) of the measured digitized states from the W-RNG *via* the ADC hardware. The ADC digital output signals are as follows: (a) *bit-8* (MSB), (b) *bit-7*, (c) *bit-6*, (d) *bit-5*, (e) *bit-4*, (f) *bit-3*, (g) *bit-2*, and (h) *bit-1* (LSB).

**6. Analysis of cross-correlation between inter devices**

Figure S5 shows the cross-correlation coefficient between inter devices *via* 8 output pins of the ADC hardware. This cross-correlation coefficient is extracted from different signals in other devices, and refers to the similarity of different signals. Because an ideal cross-correlation coefficient for perfect randomness is 1, a lower value of the cross-correlation coefficient implies that the sequence of the generated signals becomes independent of each other and less predictable. The correlation between the inter signals is extracted as follows.

$$R_{XY}(\tau)=\frac{1}{|R_{XX}(0)||R_{YY}(0)|}\int_{-\infty}^{+\infty} x\left( t \right)y(t+\tau)dt$$

All the coefficients are normalized by |*R_XX_*(0)||*R_YY_*(0)|, which is the coefficient when the time lag is zero. *R_XY_*(*τ*) ranges between -1 and 1. Herein, -1 is the perfect negative correlation and 1 is the perfect positive correlation. In contrast, the correlation of 0 implies that two variables are independent of each other, *i*.*e*., even though one variable is known, the other one is untraceable [13-14]. The procedure to extract the cross-correlation coefficient of *R_XY_*(*τ*) is described as follows. First, digital data are generated from two different devices *via* 8 output pins of the ADC hardware in the form of voltage. Second, digital bits are converted to -1 or 1 by using the switching rule: a logic value of ‘0’ is assigned as -1 and that of ‘1’ is assigned as 1. This conversion makes fair comparison possible among the cross-correlation coefficients for various *n*_ADC_ [12]. Last, the *R_XY_* is extracted as a function of the time evolution. Figure S5a to S5h plots the *R_XY_* according to the *n*_ADC_ from 8 to 1, respectively. For example, Fig. S5a shows the *R_XY_* for a *bit-8* (MSB) and Fig. S5h exhibits the *R_XY_* for a *bit-1* (LSB) signal.

The *R_XY_* of the *bit-8* signal is periodic and hardly decayed. On the contrary, the *R_XY_* of the *bit-4* signal is rapidly converged to 0, *i*.*e*., it is unpredictable because there is no relationship between the signals of different devices.

**
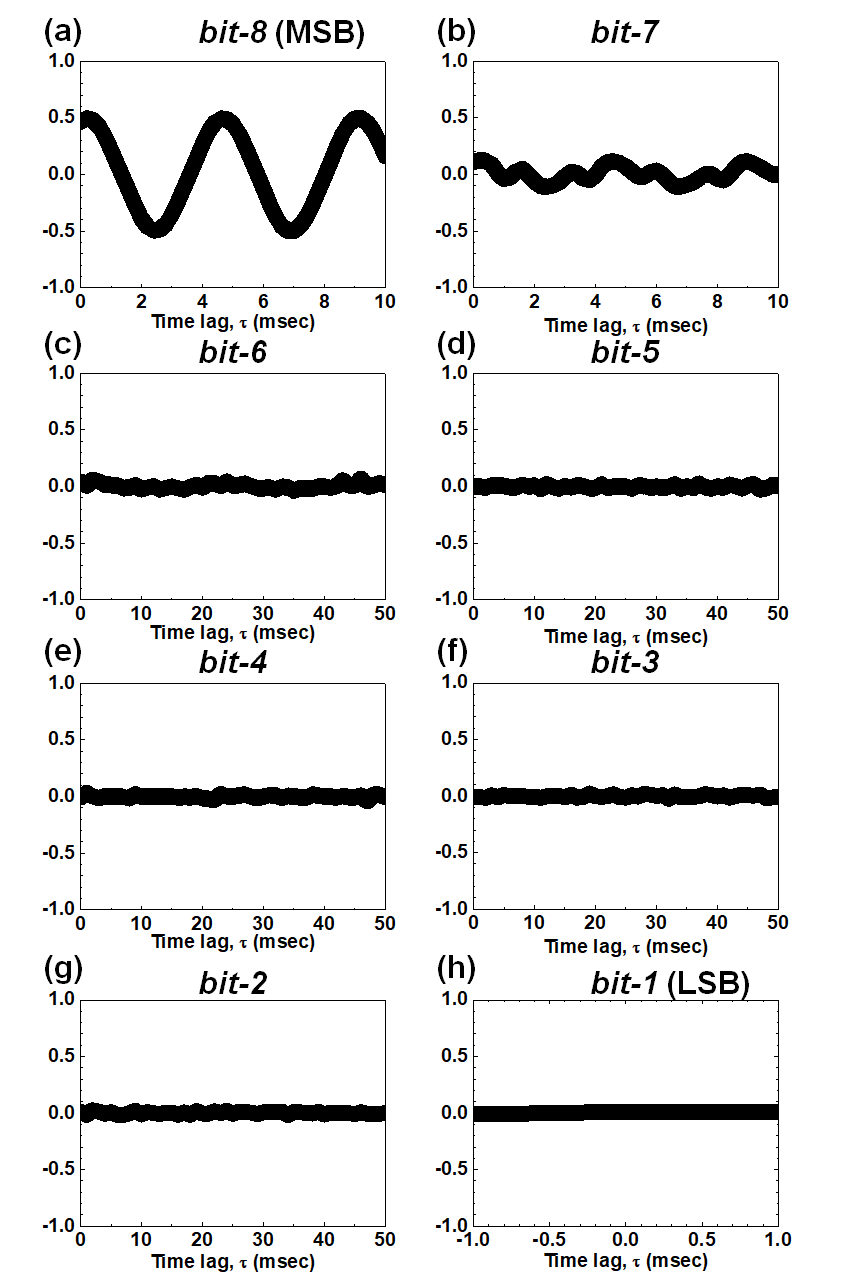
**

**Figure S5.** Cross-correlation coefficient (*R_XY_*) of the measured digitized states from different W-RNGs *via* the ADC hardware. ADC digital output signals are as follows: (a) *bit-8* (MSB), (b) *bit-7*, (c) *bit-6*, (d) *bit-5*, (e) *bit-4*, (f) *bit-3*, (g) *bit-2*, and (h) *bit-1* (LSB).

**7. Transition of digitized states by time evolution in Markov chain of digitized states for an intra device**

Figure S6 displays a contour map of the elements from the Markov chain model for an intra device in terms of time transition between two digitized states, which are represented by *P*(*X*,*Y*). When the normalized value of the *P*(*X*,*Y*) is 1, it is ideal for the uniform transition. When the *n*_ADC_ is larger than 5, the contour map for the Markov chain shows non-uniform distribution, which is denoted with multi-tones of the gray scale. In the case of *n*_ADC_ = 8, if a state at *t* = *T*_i_ is determined, another state at *t* = *T*_i+1_ is predictable. Some of the *P*(*X*,*Y*) are over 8 in the gray scale bar of Figure S6. On the contrary, when the *n*_ADC_ is smaller than 4, the contour map for the Markov chain exhibits uniform distribution, which is expressed with two-tones of the gray scale. In the case of *n*_ADC_ = 4, even though a state at *t* = *T*_i_ is known, another state at *t* = *T*_i+1_ is unpredictable. All of the *P*(*X*,*Y*) are in between 0.5 and 2 in the gray scale bar of Figure S6.

**
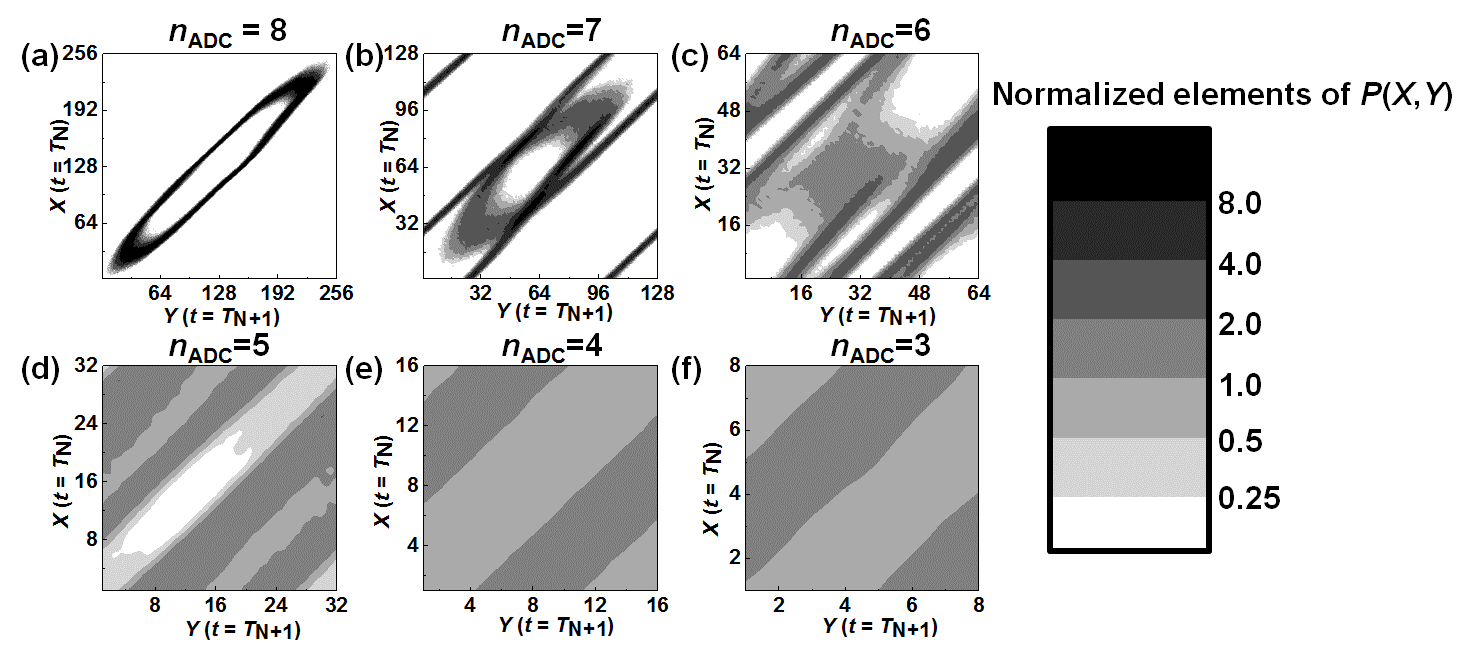
**

**Figure S6.** Contour map of the Markov chain model for an intra device in terms of time transition between digitized states according to the *n*_ADC_: (a) 8, (b) 7, (c) 6, (d) 5, (e) 4, and (f) 3.

**8. Transition of digitized states by time evolution in Markov chain of digitized states between inter devices**

Figure S7 plots a contour map of the elements from the Markov chain model for inter devices in terms of the time transition between two digitized states, which are represented by *P*(*X*,*Y*). A contour map such as this is useful to evaluate device-to-device correlation under the identical input conditions. It is crucial whether an RNG can generate unpredictable signals under identical input conditions for different devices [15-16]. When the *n*_ADC_ is smaller than 6, the contour map for the Markov chain shows uniform distribution, which is denoted with two-tones of the gray scale. All of the *P*(*X*,*Y*) are between 0.5 and 2 in the gray scale bar of Figure S7. This implies that digitized states do not possess any correlation between inter devices.

**
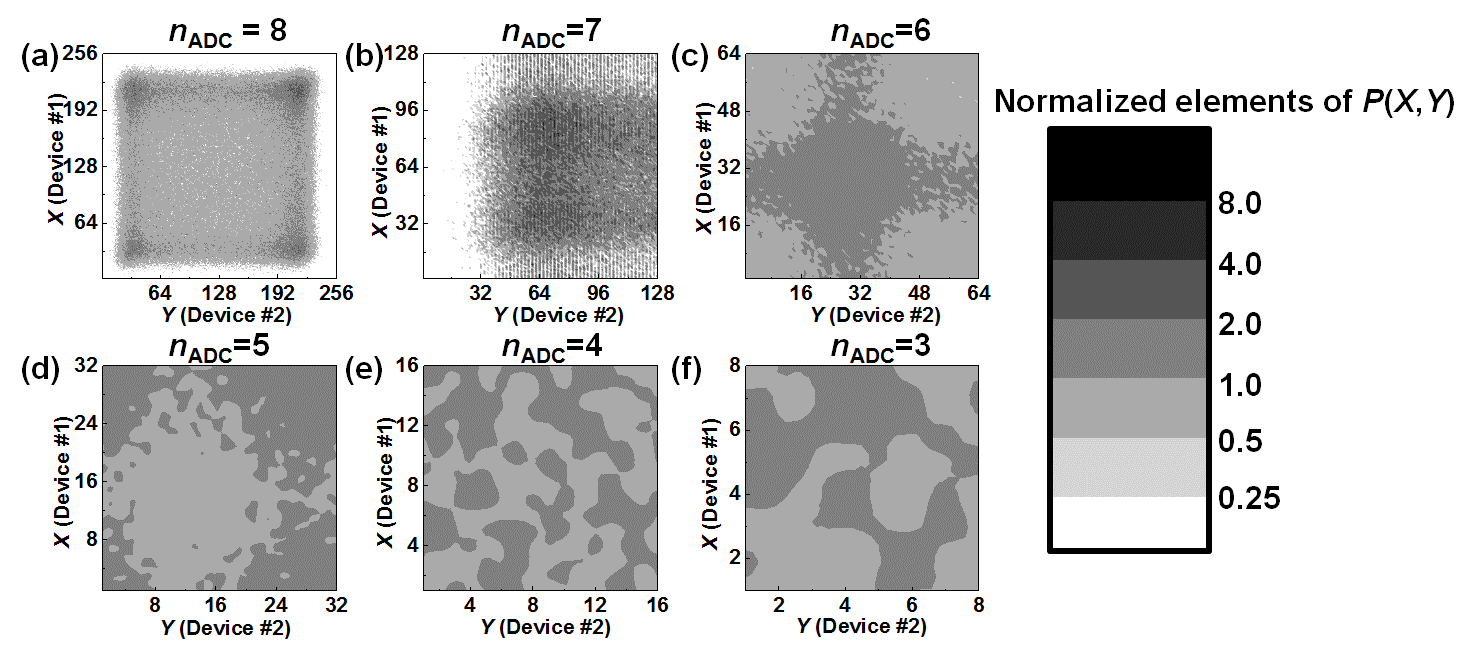
**

**Figure S7.** Contour map of the Markov chain model for inter devices in terms of time transition between digitized states according to the *n*_ADC_: (a) 8, (b) 7, (c) 6, (d) 5, (e) 4, and (f) 3.

**9. Venn diagram for joint entropy and mutual information**

Figure S8a displays a Venn diagram in terms of entropy between random variable *X* and *Y*. For an intra device, random variable *X* indicates a current state (*t* = *T*_i_), while variable *Y* refers to a next state (*t* = *T*_i+1_). For inter devices, random variable *X* indicates device #1, while variable *Y* refers to device #2. The right side of Fig. S8a exhibits geometrical configurations for (i), (iv) entropy, (ii), (v) conditional entropy, (iii) joint entropy, and (vi) mutual information, respectively. A geometrical area corresponds to an entropy value.

Figure S8b illustrates the condition for an application to a random number generator, which should possess unpredictable properties. To assure the unpredictable properties, there are two conditions in terms of joint entropy and mutual information. First, a value of joint entropy represented by *H*(*X*,*Y*) should be 2·*n*_ADC_ for *X* = *n*_ADC_ and *Y* = *n*_ADC_. Second, a value of mutual information expressed by *I*(*X*,*Y*) should be 0. Thus, the unpredictable properties are easily verified when a value of *H*(*X*,*Y*) and *I*(*X*,*Y*) meets the condition of 2·*n*_ADC_ and 0, respectively.

**
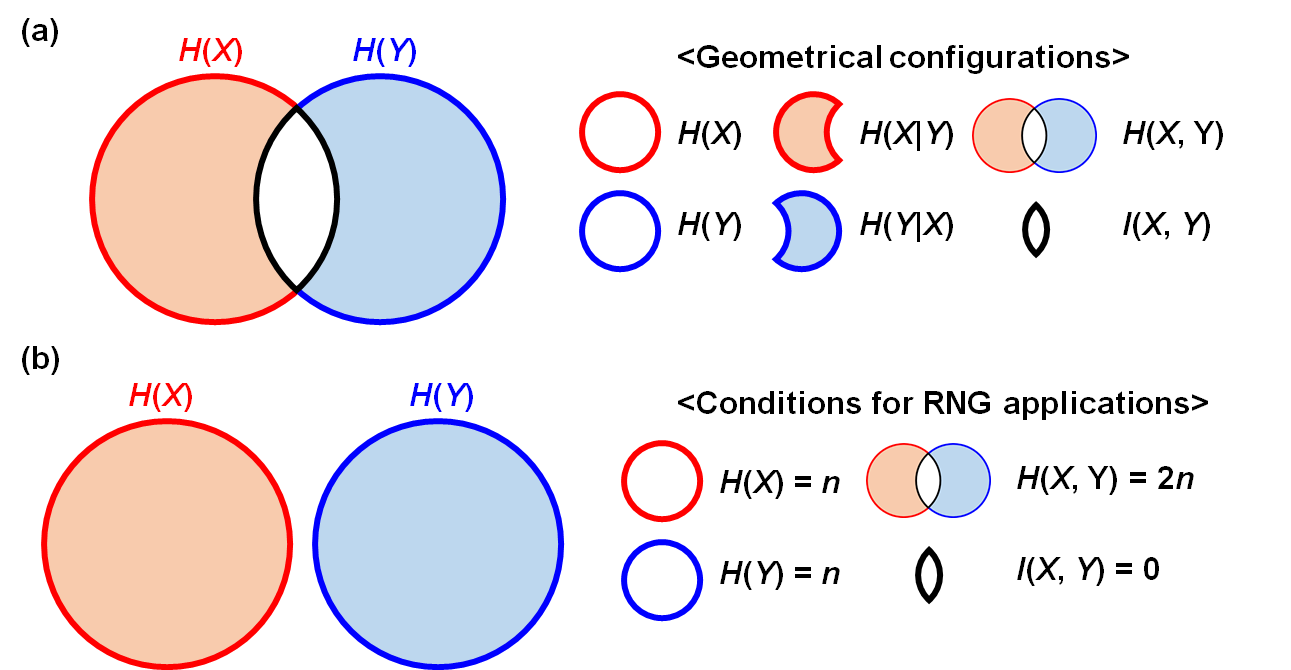
**

**Figure S8.** Graphical Venn diagram to show predictability and unpredictability. (a) Some predictability with an intersection represented by joint entropy and mutual information between random variable *X* and *Y*. (b) Unpredictability with no intersection expressed by joint entropy and mutual information between random variable *X* and *Y*.

**REFERENCES**

[1] B. Rudiyanto, B. Hariono, and A. Budiprasojo. Quadcopter Surveyor Drone Wind Velocity Data Characteristic for Optimal Hotwire Sensor Position, *Journal of Physics: Conference Series* 1569 (2020) 032096.

[2] C. MASSÉ, O. GOUGEON, D.-T. NGUYEN, and D. SAUSSIÉ, Modeling and control of a quadcopter flying in a wind field: A comparison between LQR and structured ℋ∞ control techniques, *2018 International Conference on Unmanned Aircraft Systems* (2018) 1408-1417.

[3] S. Chang, K. AlAshmouny, M. McCormick, Y. C. Chen, and E. Yoon “Bio bolt: A minimally-invasive neural interface for wireless epidural recording by intra-skin communication,” in *Proc. IEEE Symp. VLSI Circuits* (2011) 146-147.

[4] Y. Yang, J. Zhou, X. Liu, J. H. Cheong, W. L. Goh, A 151-nW adaptive delta-sampling ADC for ultra-low power sensing applications, *IEEE Transactions on circuits and systems* 63 (2016) 638-642.

[5] M. Yip and A. P. Chandrakasan, A resolution-reconfigurable 5-to-10b 0.4-to-1V power scalable SAR ADC, in *Proc. IEEE ISSCC* (2011) 190-192.

[6] G. V. Deepthi and K. N. Reddy, Design of an area-efficient and lower power 32 bit SAR ADC for biomedical applications, *Dogo rangsang research journal* 12 (2022) 19-28.

[7] P. Butter, Antialiasing filtering considerations for high precision SAR analog-to-digital converters, *Analog Dialogue* 52 (2018) 54-59.

[8] B. J. Berne, J. P. Boon, and S. A. Rice, On the calculation of autocorrelation functions of dynamical variables, *The Journal of Chemical Physics* 45 (1966) 1086-1096.

[9] X. Ma, F. Xu, H. Xu, X. Tan, B. Qi, and H. K. Lo, Postprocessing for quantum random-number generators: Entropy evaluation and randomness extraction, *Physical Review A* 87 (2013) 062327.

[10] B. H. Baltagi, S. H. Song, B. C. Jung, and W. Koh, Testing for serial correlation, spatial autocorrelation and random effects using panel data, *Journal of econometrics* 140 (2007) 5-51.

[11] F. Diaz, Performance prediction using spatial autocorrelation, *Proceedings of the 30th annual international ACM SIGIR conference on Research and development in information retrieval* (2007) 583-590.

[12] E. S. Lohan, Statistical analysis of BPSK-like techniques for the acquisition of Galileo signals, *Journal of Aerospace Computing, Information, and Communication* 3 (2006) 234-243

[13] G. F. Zebende, DCCA cross-correlation coefficient: Quantifying level of cross-correlation, *Physica A: Statistical Mechanics and its Applications* 390 (2011) 614-618.

[14] K. Plenkers, J. R. R. Ritter, and M. Schindler, Low signal-to-noise event detection based on waveform stacking and cross-correlation: Application to a stimulation experiment, *Journal of seismology* 17 (2013) 27-49.

[15] M. Kim, U. Ha, K. J. Lee, Y. Lee, and H. J. Yoo, A 82-nW chaotic map true random number generator based on a sub-ranging SAR ADC, *IEEE Journal of Solid-State Circuits* 52 (2017) 1953-1965.

[16] F. Özkaynak, Cryptographically secure random number generator with chaotic additional input, *Nonlinear Dynamics* 78 (2014) 2015-2020.
